# Genome-wide mapping of DNase I hypersensitive sites reveals chromatin accessibility changes in *Arabidopsis* euchromatin and heterochromatin regions under extended darkness

**DOI:** 10.1038/s41598-017-04524-9

**Published:** 2017-06-22

**Authors:** Yue Liu, Wenli Zhang, Kang Zhang, Qi You, Hengyu Yan, Yuannian Jiao, Jiming Jiang, Wenying Xu, Zhen Su

**Affiliations:** 1 College of Biological Sciences, China Agricultural University, State key Laboratory of Plant Physiology and Biochemistry, Beijing, China; 2 Nanjing Agricultural University, State Key Laboratory for Crop Genetics and Germplasm Enhancement, JCIC-MCP, Nanjing, China; 3 State Key Laboratory of Systematic and Evolutionary Botany, Institute of Botany, The Chinese Academy of Sciences, Beijing, China; 4 University of Wisconsin-Madison, Department of Horticulture, Madison, WI USA

## Abstract

Light, as the energy source in photosynthesis, is essential for plant growth and development. Extended darkness causes dramatic gene expression changes. In this study, we applied DNase-seq (DNase I hypersensitive site sequencing) to study changes of chromatin accessibility in euchromatic and heterochromatic regions under extended darkness in *Arabidopsis*. We generated 27 Gb DNase-seq and 67.6 Gb RNA-seq data to investigate chromatin accessibility changes and global gene expression under extended darkness and control condition in *Arabidopsis*. We found that ~40% DHSs (DNaseI hypersensitive sites) were diminished under darkness. In non-TE regions, the majority of DHS-changed genes were DHS-diminished under darkness. A total of 519 down-regulated genes were associated with diminished DHSs under darkness, mainly involved in photosynthesis process and retrograde signaling, and were regulated by chloroplast maintenance master regulators such as GLK1. In TE regions, approximately half of the DHS-changed TEs were DHS-increased under darkness and were primarily associated with the LTR/Gypsy retrotransposons in the heterochromatin flanking the centromeres. In contrast, DHS-diminished TEs under darkness were enriched in Copia, LINE, and MuDR dispersed across chromosomes. Together, our results indicated that extended darkness resulted in more increased chromatin compaction in euchromatin and decompaction in heterochromatin, thus further leading to gene expression changes in *Arabidopsis*.

## Introduction

Light is one of the essential environmental inputs for plant growth and development. Several important genes (*e.g., PHYA, PHYB, CRY2*) have been shown to be responsible for plant adaptation to light conditions^1^, and to lead to adaptive changes in cells and even the whole organism^2, 3^. Thus, the mechanism by which light affects plants has been extensively investigated. Abnormal light conditions are generally thought to induce significant phenotypic changes. For example, low red/far-red ratios of shade light induce dramatic phenotypic changes such as hypocotyl growth, petiole elongation, leaf area reduction, hyponastic leaf movement, fewer branches, and leaf senescence^3–6^. Shade light also causes early flowering, thus indicating that extended darkness might accelerate the transition from vegetative growth to reproductive growth^3, 7^. In addition, extended periods of darkness trigger leaf senescence in *Arabidopsis* ^8–10^.

Complex transcriptional networks have been shown to mediate plant development in response to light, including seedling photomorphogenesis, seed germination, shade avoidance, and photoperiod response^11^. Light modulates photoreceptor activity, which in turn triggers transcription factor (TF) regulation. For example, the phytochrome-interacting TFs, PIF4 and PIF5, negatively regulate phytochrome B (phyB)-mediated red light responses, and are required for dark-induced leaf senescence^12, 13^. The expression of the major senescence-promoting NAC transcription factor ORESARA 1 (ORE1), which is a downstream factor of *PIF4/5*, is activated in the dark^12, 13^. Under darkness, PIFs repress GOLDEN2-LIKE1 (*GLK1*), which is an important chloroplast maintenance master regulator involved in the PIFs-dependent regulatory network and influences photosynthesis-associated genes and plastid retrograde signalling^14–16^.

The regulation of various TFs may be affected by the changes in chromatin structure under different light conditions. Recently, light signalling has been reported to control nuclear architecture reorganization during establishment^17^. Changes in chromatin structure affect the binding of TFs to regulatory elements, thus altering the expression of the associated genes^18^. Consequently, DNase I hypersensitive sites (DHSs) represent chromatin regions that are accessible for TF binding and, thereby, can be used to predict the presence of TFs^19, 20^. DHS mapping analysis has been broadly performed for the global identification of cis-regulatory elements (CREs) and TF binding sites^21–24^. Currently, DHSs are known to be closely associated with gene expression in different species, such as *Arabidopsis* ^24^, rice^25^, human^21, 23^, and other species. DNase-seq (DNase I treatment coupled with high-throughput DNA sequencing) is a powerful technique for identifying cis-regulatory elements across the genome and is highly effective for comparing TF binding profiles in different development stages and environmental conditions. For example, Sullivan *et al*. have conducted DNase-seq against of 7-day-old seedlings and have uncovered light-cued regulatory DNA dynamics and TF network remodeling during photomorphogenesis^26^.

Moreover, extended darkness has been reported to cause dramatic changes in gene expression in *Arabidopsis*. A large number of differentially expressed genes (DEGs) under extended darkness and control condition in *Arabidopsis* have been identified using ATH1 GeneChip or large-scale quantitative real-time PCR (qRT-PCR)^8, 27, 28^. Lin and Wu have examined global gene expression in response to 1-day and 5-day dark treatment by ATH1 GeneChip^8^. Parlitz *et al*. revealed the differential expression of senescence-associated genes and TFs after 2-day and 4-day dark treatment by qRT-PCR^27^. We have also performed ATH1 GeneChip-based transcriptional profiling data for extended darkness treatment^28^. Integration analysis of gene expression datasets generated from different laboratories has demonstrated that the DEGs have ~70% or even more overlap across different days of extended darkness (Supplementary Fig. 1), thus indicating that DEGs at different time-points after exposure to extended darkness are highly consistent.

To study the possible regulatory elements of DEGs under extended darkness in *Arabidopsis*, we conducted DNase-seq and RNA-seq to study chromatin accessibility and the association between DHSs and gene expression in *Arabidopsis* plants under extended darkness and control conditions. We also used combinatorial analyses of DNase-seq and sRNA-seq to study the relationship between DHSs and siRNA changes in transposable element (TE) regions under these two conditions.

## Results

### Genome-wide mapping of DNase I hypersensitive sites under extended darkness treatment and control condition

**Figure 1:**
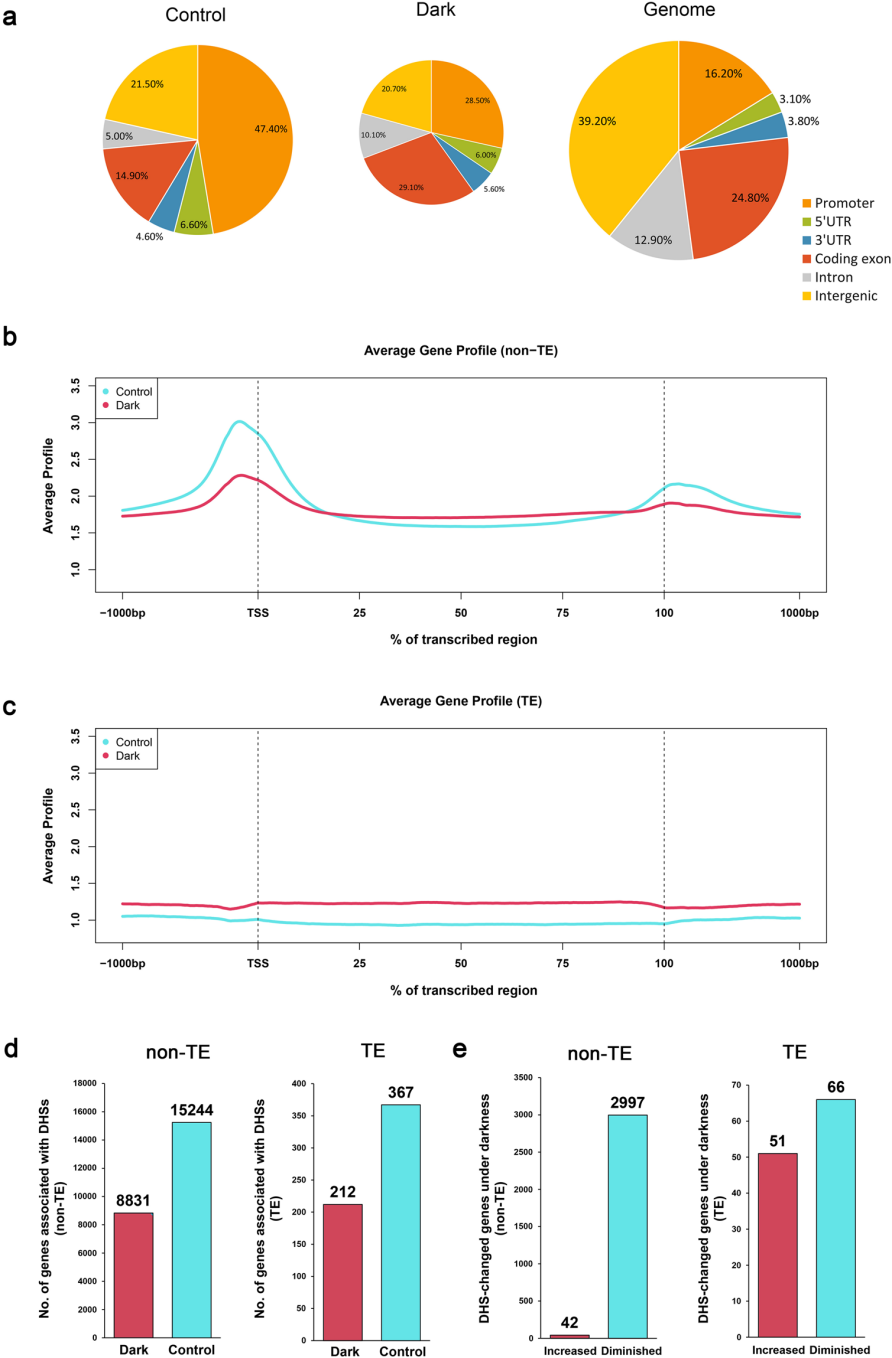
Distribution of DHSs under extended darkness and control condition. (**a**) Percentage of DHSs within different regions of the *Arabidopsis* genome under extended darkness and control conditions. The genome was divided into six classes: promoter, 5′UTR, 3′UTR, coding exon, intron and intergenic regions. The “Genome” panel represents the proportion of these six classes in *Arabidopsis* genome. (**b**,**c**) The profile of the DHS distribution in all *Arabidopsis* non-TE (**b**) and TE (**c**) genes including 1 kb upstream and downstream under both extended darkness and control conditions. A meta-gene profile was generated using the normalized sequencing density of DHSs. The X-axis represents the conversion of the gene body into a percentage to standardize genes of different lengths. The 1-kb upstream and downstream regions of the gene are included. The Y-axis represents average values of DNase hypersensitivities. (**d**) Number of non-TE and TE genes associated with DHSs under darkness and light. (**e**) Number of DHS-changed non-TE and TE genes under darkness and light.

Little is known about chromatin dynamics in response to extended darkness, despite the significant number of differentially expressed genes under extended darkness and normal conditions. Changes in chromatin accessibility affect TF binding to regulatory elements, thereby affecting the expression of the associated genes^18^. To investigate chromatin dynamics under extended darkness and control condition, we performed DNase-seq using 15-day-old *Arabidopsis* whole plants exposed to 3-day darkness treatment with normal light condition as a control. We identified a total of 10,380 DHSs and 5,948 DHSs under control and darkness conditions, respectively (Supplementary Table 1). Forty percent (40%) of the DHSs were diminished after dark treatment, thus indicating that the chromatin might have been condensed. We examined the genome distribution of the DHSs under each condition. We divided the *Arabidopsis* genome into six subclasses: promoter, 5′UTR, 3′UTR, coding exon, intron, and intergenic regions. The distributions of the DHS locations relative to *Arabidopsis* genes differed between the two conditions (Fig. 1a). Generally, a higher percentage of DHSs were present in promoter regions than in other genomic regions, in agreement with previous findings showing that promoters are associated with open chromatin in mammalian and plant genomes^21, 24, 25^. However, the percentage of DHSs located in promoter regions under darkness decreased by 18.9% compared with that under control condition (28.5% vs. 47.4%). In addition, exon regions occupied 29.1% under darkness, compared with 14.9% under control condition.

DNA sequences in chromatin include TEs and non-TEs. TEs can change their positions within a genome. The regulation of TEs may differ from that of protein-coding genes. To investigate the effect of extended darkness on chromatin changes in both TE and non-TE genes, we divided DHSs into non-TE-related DHSs and TE-related DHSs. Meta-gene profiles of the DHSs generated along the generic region of non-TE genes showed that the profile of the DHS distribution upstream of transcription start sites (TSSs) was similar under both conditions (Fig. 1b), but the signal intensities of DHSs were higher under control condition than under darkness, especially for DHSs located in promoter regions near TSSs. Thus, extended darkness might induce chromatin condensation and decrease chromatin accessibility near promoter regions. However, the distribution of TE-related DHSs displayed very different patterns (Fig. 1c). The signals for DNase I sensitivity in TE regions were higher under extended darkness than under control condition. Under dark treatment, DHSs preferentially localized to TE bodies, in contrast to the distribution of non-TE genes.

To identify non-TE genes associated with DHSs under darkness and control conditions, we used CEAS software^29^ to statistically analyse DHSs located within the 3 kb upstream or 1 kb downstream of TSSs, which were defined as DHS-associated genes. We identified 15,244 non-TE genes under control condition and 8,831 non-TE genes under darkness (Fig. 1d). The number of DHS-associated genes significantly decreased under darkness. Collectively, the DHS characterization revealed that changes in chromatin accessibility are associated with genic regions under extended darkness. To further investigate differences in chromatin accessibility between the two conditions, we identified differential DHS regions (by using MACS) located in the regions 3 kb upstream and 1 kb downstream of TSSs as DHS-changed genes. We identified 42 DHS-increased and 2,997 DHS-diminished non-TE genes under extended darkness (Fig. 1e and Supplementary Data 1), thus suggesting that darkness might decrease chromatin accessibility within non-TE regions. Meanwhile, we identified 367 and 212 TE-related genes under normal condition and extended darkness, respectively (Fig. 1d). We further identified 51 DHS-increased and 66 DHS-diminished TE genes under extended darkness (Fig. 1e and Supplementary Data 2). We also used public DNase-seq data (GSM847326-28, 2-week-old seedling plants) as a control to verify the DHS-changed genes under extended darkness (Supplementary Data 3), and approximately 76.6% of all the DHS-changed genes (including TE and non-TE genes) were confirmed.

To understand the functions of these DHS-changed genes, gene ontology (GO) enrichment analysis of 2,997 DHS-diminished genes was conducted by using agriGO^30^ and REVIGO^31^ (Supplementary Fig. 2 and Supplementary Table 2). The GO term associated with the regulation of transcription was significantly enriched, including *MYB*, *WRKY*, *NAC* family genes. DHS-diminished genes were strongly associated with the GO terms of photosynthesis and light, response to stimulus and stress, and hormone-mediated signaling pathway. GO terms of anatomical structure development, such as leaf morphogenesis, epidermal cell differentiation, cell cycle and cytoskeleton organization, were also enriched. Interestingly, GO terms such as gene silencing and ncRNA metabolic process were significantly enriched in these DHS-diminished genes under extended darkness.

### Differential chromatin accessibility and transcriptional activity of non-TE genes under extended darkness and control condition

To investigate the relationship between DHSs and gene expression, we conducted RNA-seq using the same materials as those used for the DNase-seq experiments with three independent biological replicates (Supplementary Table 3). Differentially expressed genes under extended darkness and control condition were identified using Cufflinks software^32^ (Supplementary Data 4). In total, we identified 4,259 genes with significantly altered expression (fragments per kilobase of transcript per million mapped reads [FPKM] value > 2-fold change, p-value < 0.05), including 2,111 up-regulated genes and 2,148 down-regulated genes, after dark treatment (Supplementary Fig. 3a). To verify the RNA-seq results, we selected some genes for real-time RT-PCR validation. The additional biological samples were collected under the same conditions used for the RNA-seq experiments. The real-time RT-PCR results for the majority of the selected genes confirmed the RNA-seq results (Supplemental data 5). Moreover, we also compared our RNA-seq data with the published ATH1 GeneChip results^8, 28^ under extended darkness (Supplemental data 4). Among the 4,259 changed genes, more than 70% genes were also detected with similar trends in previously published microarray results.

To understand the involvement of these DEGs in biological processes and functions, we conducted GO enrichment analysis by using agriGO^30^ and REVIGO^31^ (Supplementary Fig. 3b,c and Supplementary Tables 4,5). As expected, under extended darkness, the GO terms response to absence of light and shade avoidance were significantly enriched; in contrast, under the control condition, photosynthesis and light response related GO terms were highly enriched. In addition, GO terms associated with anatomical structure development and hormone-mediated signal pathways were also enriched under the control condition. The most significant GO terms were associated with transcriptional regulation under both extended darkness and control condition, a result consistent with previous findings in which TFs have been found to be differentially expressed in response to dark treatment^8, 27^.

**Figure 2:**
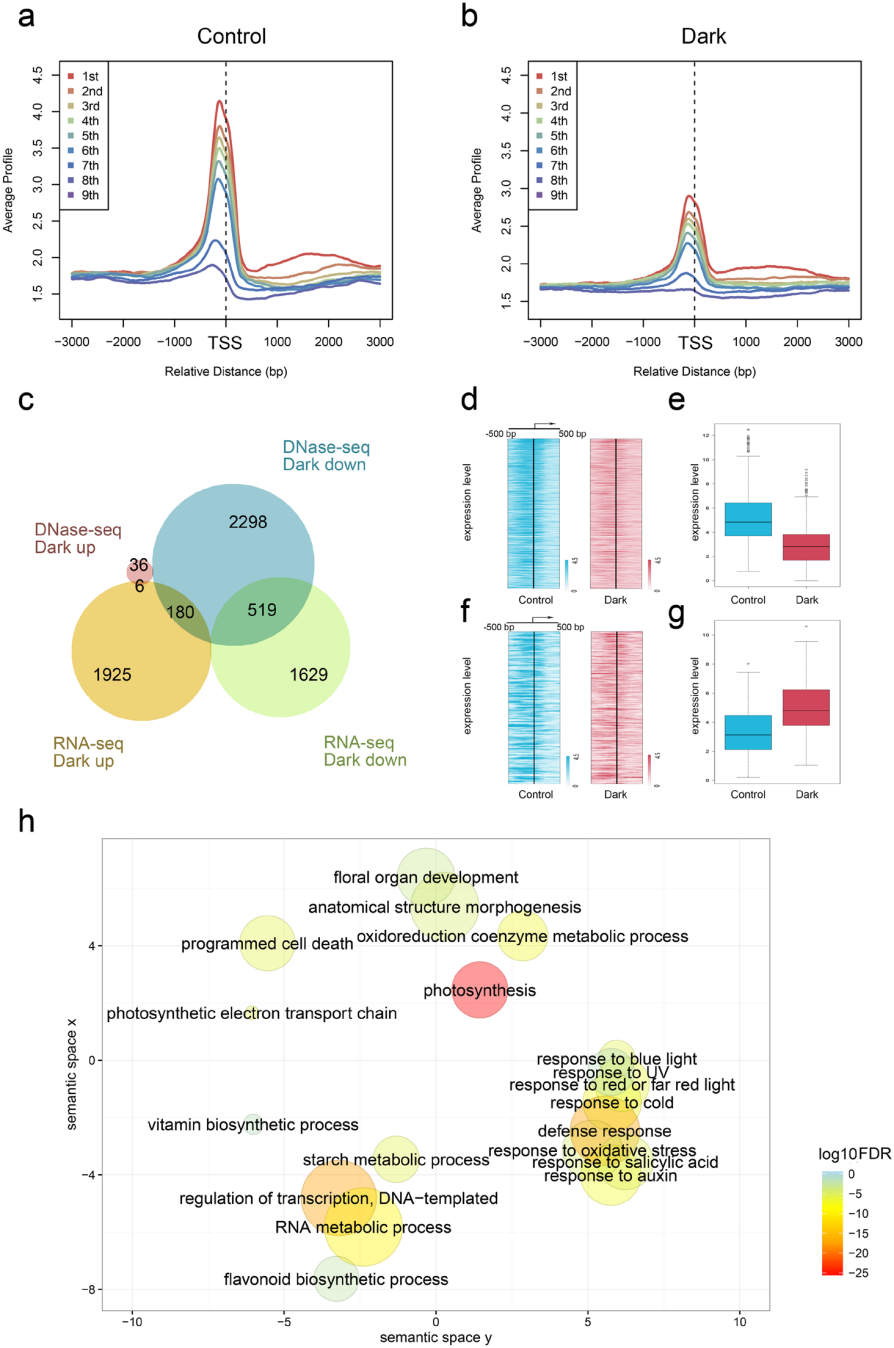
Integrated analysis of DHS-changed and differentially expressed genes. (**a**,**b**) The distribution of DHSs along 3 kb upstream and downstream of TSSs of *Arabidopsis* genes with different expression level under control (**a**) and extended darkness (**b**), respectively. All expressed genes were divided into 9 bins, which were expressed genes (FPKM > 0) from high expression (1st) to low expression (9th) based on the expression levels. (**c**) Venn diagrams between DHS-changed genes and differentially expressed genes under extended darkness. (**d**) DHS intensities around TSSs of the group of 519 genes from (**c**) under extended darkness and control conditions, respectively. The genes were sorted according to the expression level determined by FPKM under control condition. For each gene, the DHS intensities are displayed along −500 bp to 500 bp regions around the TSS. (**e**) The gene expression values are shown for the group of 519 genes from (**c**). (**f**) DHS intensities around TSSs of the group of 180 genes from (**c**) under extended darkness and control conditions, respectively. The genes were sorted according to the expression level determined by FPKM under control condition. For each gene, the DHS intensities are displayed along −500 bp to 500 bp regions around the TSS. (**g**) The gene expression values are shown for the group of 180 genes from (**c**). (**h**) GO enrichment analysis of 519 genes in (**c**) by agriGO and REVIGO. The scatter plot shows the cluster representatives in a two-dimensional space derived by applying multidimensional scaling to a matrix of the significant GO terms with semantic similarities. Bubble color and size indicates the log_10_(FDR p-value) (legend in bottom right-hand corner).

We integrated DNase-seq with RNA-seq datasets under darkness and control condition together. Genes with higher expression displayed higher levels of DNase I sensitivity under both darkness and normal conditions (Fig. 2a,b). Next we compared the groups of DHS-changed genes and differentially expressed non-TE genes, and discovered that most (98.6%) of DHS-changed genes were DHS-diminished genes under extended darkness treatment (Fig. 2c). There were 519 DHS-diminished and down-regulated genes (Fig. 2d,e and Supplementary Data 6) under extended darkness, thereby indicating a positive relationship between DNase I hypersensitivity and gene expression for these 519 genes. In contrast, there were 180 DHS-diminished genes (Supplementary Data 7) under extended darkness treatment showing negative relationship between DNase I hypersensitivity and gene expression, with lower DNase I hypersensitivity values (Fig. 2f) and higher FPKM values (Fig. 2g). This antagonistic relationship suggested that other regulatory mechanisms such as non-coding RNAs, may be involved in the differential gene expression under extended darkness and control condition.

To gain insight into the potential functions of these 519 DHS-diminished genes under extended darkness, we performed GO enrichment analysis (Fig. 2h and Supplementary Table 6) and found that photosynthesis (FDR P-value = 1.5E-26) was the most enriched GO term. Some processes closely related to photosynthesis, such as photosynthetic electron transport chain (FDR P-value = 3.00E-06), were also significantly enriched. GO terms associated with response to light, such as red light and far-red light (FDR P-value = 5.90E-07), blue light (FDR P-value = 4.00E-06), and UV (FDR P-value = 0.03) were enriched. In addition to light-related GO terms, development-related GO terms such as floral organ development (FDR P-value = 0.00077), as well as hormone-related terms such as salicylic acid biosynthetic process (FDR P-value = 9.00E-06), were also enriched. In addition to GO enrichment analysis, we performed gene sets enrichment analysis (GSEA) of gene family, PlantCyc and KEGG by using PlantGSEA^33^. Significant gene sets were all associated with photosynthesis and some closely related gene sets such as chlorophyll and starch (Table 1). We compared the enriched GO terms for these 519 DHS-diminished and down-regulated genes with those of all DHS-diminished genes and all down-regulated genes (Supplementary Data 8). All three groups were enriched in photosynthesis-and light-related GO terms. There were relatively more development-related GO terms enriched in the group of all DHS-diminished genes and the group of all down-regulated genes. Other GO terms, such as gene silencing, ncRNA processing, cytoskeleton organization and cell cycle process, tended to enriched in the group of the all DHS-diminished genes. We also analysed GO enrichment for those 180 genes with a negative association between DHSs and expression (Supplementary Table 7). The regulation of transcription (FDR P-value = 2.3E-12) was mostly enriched, but the GO terms related to light were not enriched in these 180 genes.

**Table 1:** Enriched gene sets among DHS-diminished and down-regulated genes under extended darkness.

Gene Set Name	No. Genes	No. Genes in the Overlap	FDR
**Gene Family**			
Chloroplast and Mitochondria gene families,Chlorophyll a/b-binding protein family	17	6	1.57E-03
**PlantCyc**			
photosynthesis light reactions	38	11	5.41E-07
starch degradation	25	6	5.33E-03
**KEGG**			
Photosynthesis - antenna proteins	19	7	3.01E-05
Photosynthesis	73	11	3.01E-05
Starch and sucrose metabolism	88	11	1.21E-04
One carbon pool by folate	15	4	0.0196
Carbon fixation in photosynthetic organisms	75	7	0.034

### Identification of regulatory elements for DHS-diminished and down-regulated photosynthesis-associated genes under extended darkness

As described above, the 519 DHS-diminished and down-regulated genes included many photosynthesis-associated genes. We found that these genes were involved in different biological processes related to photosynthesis, such as photosynthetic electron transport chain, light harvesting, light reaction, chloroplast organization, and chlorophyll metabolic process (Supplementary Data 9). We also found some genes involved in retrograde signaling (RS), such as GUN4, GUN5, and CH1.

**Figure 3:**
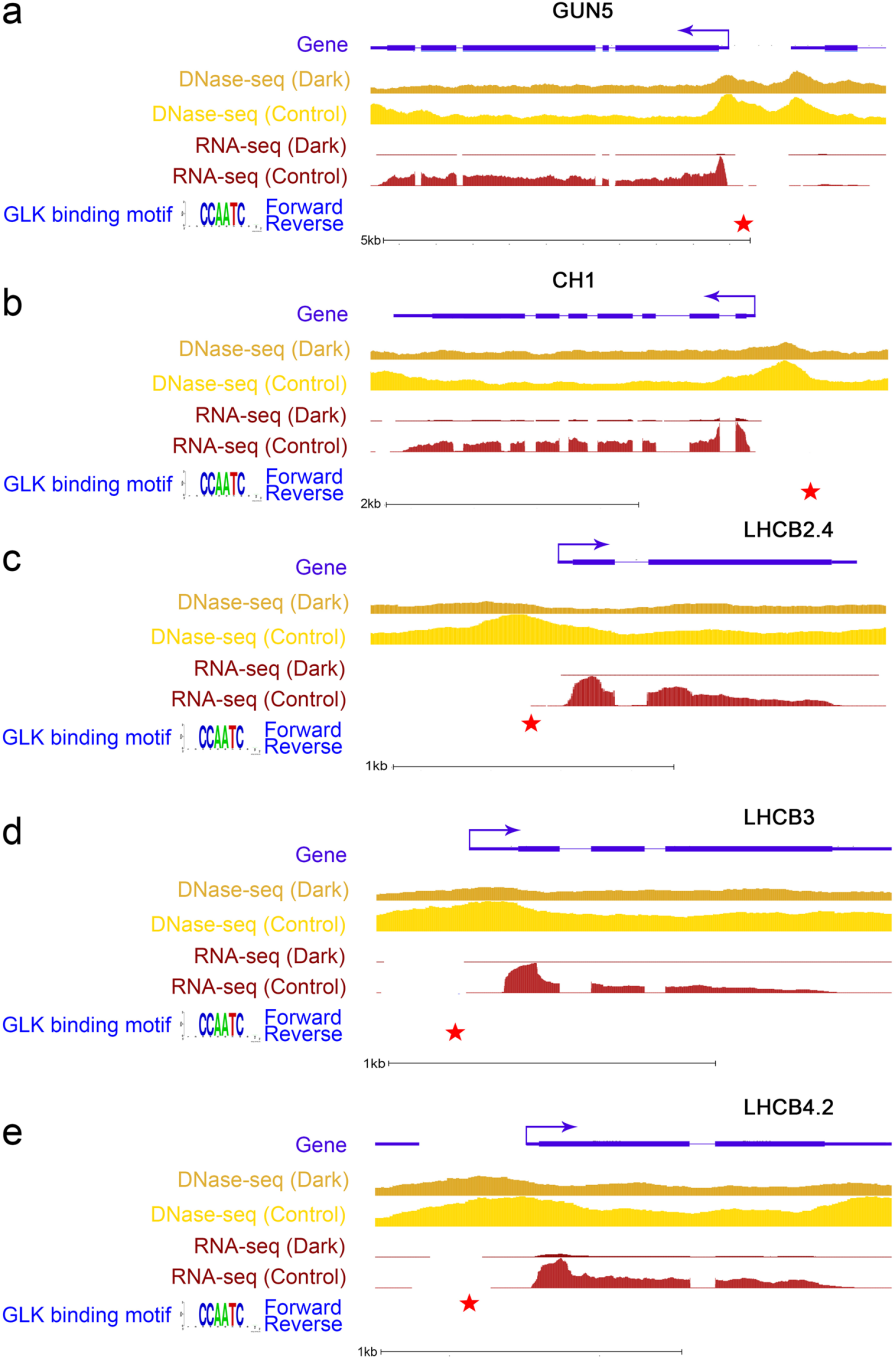
Selected genes associated with DHSs, expression and motifs in the UCSC genome browser. The gene models are shown in purple, with the direction marked by an arrow. The brown and yellow colors represent DHS signals under extended darkness and control condition, respectively. The red five-pointed stars represent the location of motifs. The dark red and light red colors represent expression signals under extended darkness and control condition, respectively. (**a**) *GENOMES UNCOUPLED 5 (GUN5)* encodes a magnesium chelatase involved in plastid-to-nucleus signal transduction. (**b**) *CHLORINA 1 (CH1)* encodes chlorophyllide a oxygenase which converts chlorophyllide a to chlorophyllide b by catalysing two successive hydroxylations at the 7-methyl group of chlorophyllide a. (**c**) *LIGHT-HARVESTING CHLOROPHYLL B-BINDING 2 (LHCB2.4)* belongs to the Lhc supergene family encodes the light-harvesting chlorophyll a/b-binding (LHC) proteins that constitute the antenna system of the photosynthetic apparatus. (**d**) *LIGHT-HARVESTING CHLOROPHYLL B-BINDING PROTEIN 3* (*LHCB3*) is a component of the main light-harvesting chlorophyll a/b-protein complex of Photosystem II (LHC II). (**e**) *LIGHT HARVESTING COMPLEX PHOTOSYSTEM II* (*LHCB4.2*) is a protein involved in the light-harvesting complex of photosystem II. The mRNA has cell-to-cell mobility.

**Figure 4:**
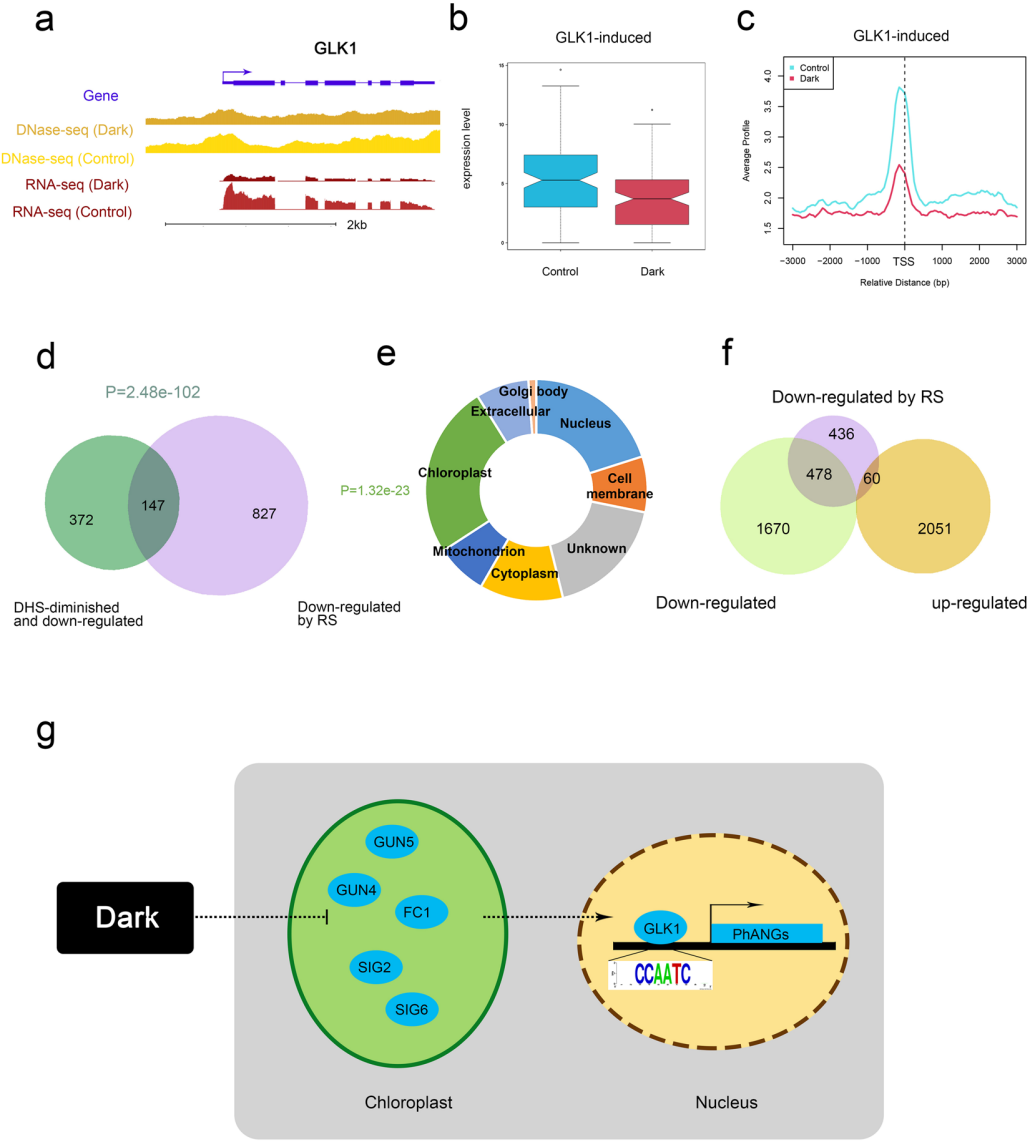
Changes in genes involved in retrograde signaling under extended darkness. (**a**) DNase-seq and RNA-seq tracks indicate a gene model for GLK1 in the UCSC genome browser. The gene models are shown in purple, with the direction marked by an arrow. The brown and yellow colors represent DHS signals under extended darkness and control condition, respectively. The dark red and light red colors represent that expression signals under extended darkness and control condition, respectively. (**b**) Expression changes in GLK1-induced genes under extended darkness. (**c**) DHS changes in GLK1-induced genes under extended darkness. (**d**) Venn diagram showing down-regulated genes by RS and 519 down-regulated and DHS-diminished genes under extended darkness. The P-value for the overlap in these 519 genes (hypergeometric distribution) is shown at the top. (**e**) Subcellular location of 519 DHS-diminished and down-regulated genes. The P-value for chloroplast in these 519 genes (hypergeometric distribution) is shown to the left. (**f**) Venn diagram showing down-regulated genes by RS and up- or down-regulated under extended darkness. (**g**) A model for darkness-influenced retrograde signaling. Darkness can repress some genes in chloroplasts in term of expression or diminished DHS, which regulate GLK1 binding to conserved motifs in the promoters of PhANGs to promote gene expression by retrograde signaling.

During motif analysis of the differential DHSs around photosynthesis-associated genes within the 519 DHS-diminished and down-regulated genes, we also found that in the group associated with photosynthesis, the putative GLK-binding site “CCAATC”^16^ was significantly enriched (Z-score = 2.22; P-value = 0.01) (Supplementary Table 8). According to previous studies, GLK1, as a chloroplast maintenance master regulator, is involved in a complex regulatory network during darkness^15^. To further elucidate the relationships among DHSs, gene expression, and motif positions, we used the UCSC genome browser^34^ to analyse GUN5, CH1, LHCB2.4, LHCB3 and LHCB4.2 involved in photosynthesis (Fig. 3). The GLK binding motif and surrounding regions were located in the promoter regions of these down-regulated photosynthesis-associated genes under darkness, which were also the regions with lower DNase I hypersensitivity under darkness. Our RNA-seq analysis showed that GLK1 was down-regulated under extended darkness (Fig. 4a). We analysed the GLK1-induced genes reported previously^16^ together with our identified DHSs and gene expression levels. These GLK1-induced genes displayed lower expression and lower DNase I sensitivities under darkness (Fig. 4b,c). Our results indicated that extended darkness might block GLK1 binding sites and further repress the expression of photosynthesis-related genes.

*GLK1* has been reported to be a crucial factor regulating photosynthesis-associated nuclear genes (PhANGs) expression in retrograde signalling^35, 36^. Dark-grown seedlings undergoing photomorphogenesis have recently been reported to resembl seedlings grown on lincomycin (a drug affecting retrograde signaling by inhibiting chloroplast biogenesis) and norflurazon (an herbicide that inhibits carotenoid biosynthesis, thereby influencing retrograde signaling)^37^. Analyzing of the changes in DHSs and expression of genes involved in retrograde signaling^35^, revealed that the related genes tended to be down-regulated and DHS-diminished under extended darkness, especially *GUN4*, *GUN5* and *FC1*, which are involved in chloroplast biogenesis, as well as *SIG2* and *SIG6*, which are important components of chloroplast transcription (Supplementary Table 9). The *gun4*, *gun5*, and *sig6* mutants showed lower chlorophyll accumulation than did wild type^38, 39^. To study the influence of DHS on retrograde signaling under extended darkness, we found that down-regulated genes associated with retrograde signaling^37^ were significantly enriched among these 519 genes under extended darkness (Fig. 4d). Additionally, among these 519 genes, those located in chloroplasts were significantly enriched (Fig. 4e). Furthermore, we found that more than half of the down-regulated genes related to chloroplast retrograde signaling^37^ overlapped with those that were down-regulated under extended darkness (Fig. 4f). Together with the changes in genes involved in retrograde signaling, we speculated that darkness influenced retrograde signaling by down-regulating *GUN4, GUN5, FC1, SIG2* and *SIG6* in chloroplasts, thereby repressing the transcriptional regulation of PhANGs by GLK1 (Fig. 4g).

### Identification of DHSs in different TE families and related siRNAs under extended darkness and control condition

A total of 367 and 212 TE-related genes were identified under normal condition and extended darkness, respectively, including 51 DHS-increased and 66 DHS-diminished TE genes under darkness (Fig. 1e and Supplementary Data 2). The TE families differed in terms of DHSs under extended darkness and control condition (Table 2). The DHS-diminished TEs under extended darkness belonged primarily to the LTR/Copia, LINE/L1 and DNA/MuDR families, which were located dispersedly on chromosomes^40^. By contrast, most of the DHS-increased TEs were LTR/Gypsy retrotransposons located in the heterochromatin flanking the centromeres^40^ (Supplementary Fig. 4). This finding indicated that chromatin tended to loosen in heterochromatin under extended darkness, in agreement with results from previous studies showing that light signalling affects chromatin organization^17^.

**Table 2:** Differential TE families in DHSs under extended darkness and control condition.

type	DHS-increased under extended darkness		DHS-diminished under extended darkness		Genome	
	count	percentage	count	percentage	count	percentage
LTR/Gypsy	37*	72.55%*	5	7.58%	1277	32.65%
LTR/Copia	4	7.84%	18*	27.27%*	514	13.14%
LINE/L1	2	3.92%	19*	28.79%*	398	10.18%
DNA/MuDR	1	1.96%	17*	25.76%*	725	18.54%
DNA/HAT	0	0.00%	1	1.52%	84	2.15%
DNA/En-Spm	3	5.88%	2	3.03%	356	9.10%
DNA/Harbinger	0	0.00%	0	0.00%	43	1.10%
RC/Helitron	0	0.00%	1	1.52%	166	4.24%
null/SADHU	0	0.00%	1	1.52%	17	0.43%
Unassigned	4	7.84%	2	3.03%	326	8.34%

*Indicates a significant difference. Significance was calculated by hypergeometric distribution with a cut-off of 0.05.

Transposons have been reported to become activated in response to environmental stress through the repression of gene silencing, such as via RNA-directed DNA methylation (RdDM)^41^. As described above, the GO terms of gene silencing and ncRNA metabolism process were significantly enriched in 2997 DHS-diminished genes. Subsequently, we analysed changes in expression and DHS among genes involved in the RdDM pathway under extended darkness. We found that the genes involved in RdDM tended to be down-regulated and/or DHS-diminished under extended darkness (Supplementary Table 10), including *AGO4*, *DMS3*, and *NRPD1A*. Thus, we speculated that the RdDM pathway might be influenced by extended darkness treatment.

TEs are closely related to small interfering RNAs (siRNAs), in that they are transcribed into non-protein-coding transcripts that are immediately converted into double-stranded RNA^41, 42^. These short double-stranded RNAs are cleaved into siRNAs involved in siRNA-mediated gene silencing. Therefore, we studied the changes in siRNA under extended darkness and control condition via sRNA-seq using the same materials as those were used for the RNA-seq and DNase-seq experiments (Supplementary Table 11). The siRNAs associated with TEs were mainly 21- to 24-nucleotide siRNAs, and the length distribution of the sequenced small RNAs was investigated^41, 42^. Our results showed that 21–24 nt sRNA had relatively high abundance, especially 21 nt and 24 nt sRNAs (Supplementary Fig. 5).

**Figure 5:**
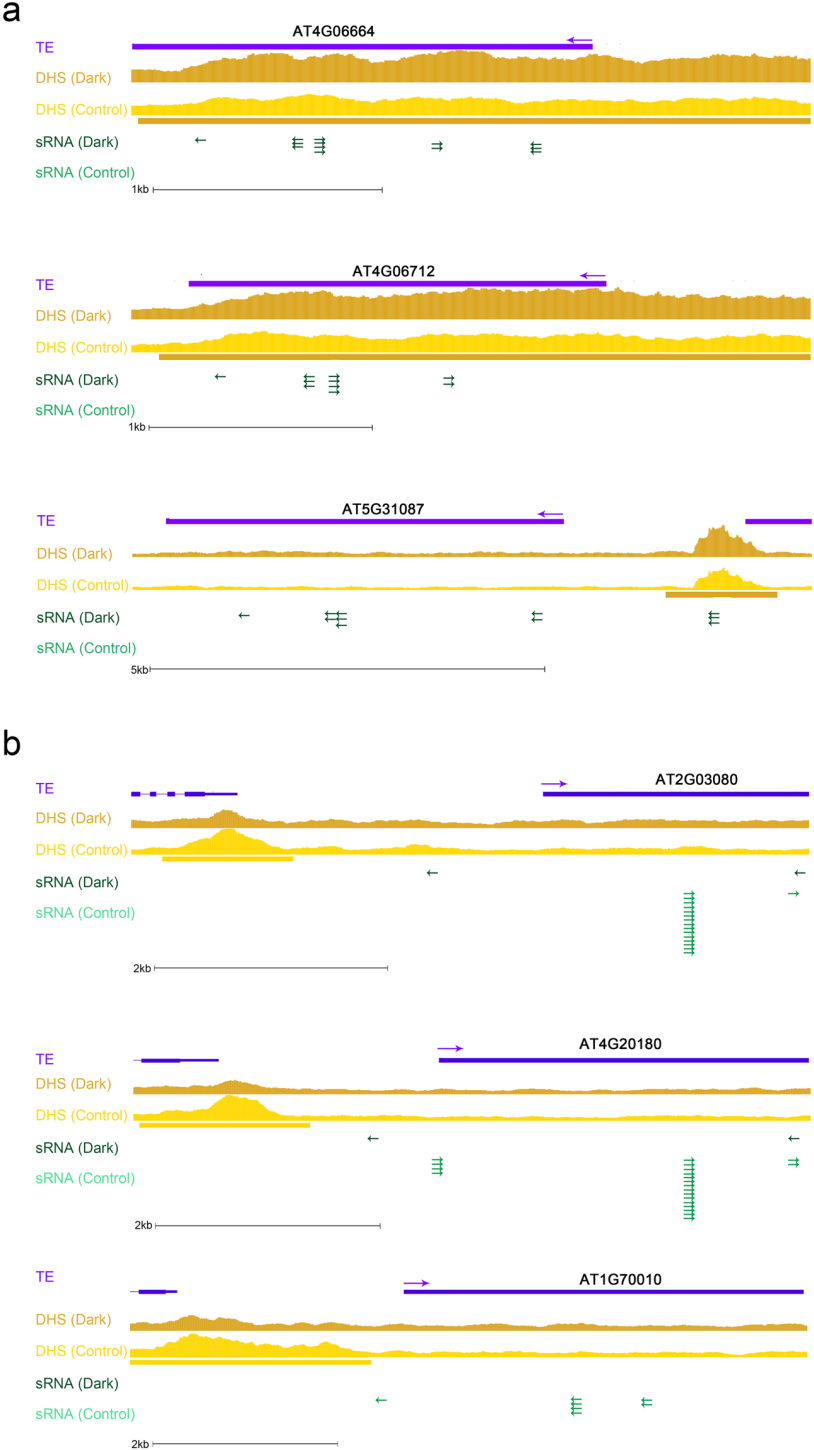
Selected TEs associated with DHSs and mapped siRNA reads in the UCSC genome browser. The gene models are shown in purple, with the direction marked by an arrow. The brown and yellow colors represent that DHS signals under extended darkness and control condition, respectively. The DHS-increased region is marked by a brown bar, and the DHS-diminished region is marked by a yellow bar. The dark and light green colors represent siRNA reads. The direction of mapping is marked by an arrow. (**a**) siRNA-increased Gypsy-like TEs. (**b**) siRNA-decreased Copia-like TEs.

We identified candidate siRNA reads by mapping them to repeat regions for subsequent analysis and then overlapping the siRNA reads with the location of the TEs. These siRNAs were found to be located in 974 and 1031 TE regions under control and extended darkness conditions, respectively. We then identified siRNA-changed TEs based by calculating reads per million (RPM) for one TE (fold change ≥ 1.6 and *P* < 0.05), respectively. We identified 183 siRNA-increased and 131 siRNA-decreased TEs under darkness (Supplementary Data 10). Furthermore, the families of siRNA-changed TEs were very similar to dynamically changed TEs in DHSs (Supplementary Table 12). For example, the siRNA-increased TEs under extended darkness mainly belonged primarily to the Gypsy families. As shown in Fig. 5, some siRNA-changed TEs were DHS-changed under extended darkness and control condition. The siRNA-increased Gypsy-like TEs such as *AT4G06664, AT4G06712* and *AT5G31087* were also increased in DHSs in their promoter or gene regions, whereas siRNA-decreased Copia-like TEs such as *AT2G03080*, *AT4G20180* and *AT1G70010* were diminished in DHSs upstream of TSSs. Thus, we proposed that differential DHSs in TE regions are strongly associated with siRNA changes under extended darkness and control condition.

## Discussion

### Influence of extended darkness on chromatin accessibility

Extended darkness causes dramatic changes in transcriptional activity, which is controlled by light-responsive *cis*-elements. DNase I hypersensitivity is an efficient method for charting chromatin accessibility and DHSs in chromatin, and can further be used to map functional elements. To study transcriptional regulation, we conducted DNase-seq analysis under extended darkness and control condition in mature *Arabidopsis* plants. Through genome-wide mapping of chromatin accessibility, we revealed the chromatin dynamics from control condition to extended darkness. We identified a total of 10,380 DHSs corresponding to control condition, and 5,948 DHSs under extended darkness. Approximately 40% of the DHSs were diminished after extended darkness, thus indicating that chromatin might become compacted after extended dark treatment. Genomic distribution analysis of the DHSs under both conditions showed that in the promoter regions, chromatin under control condition appeared to be more accessible than that under darkness, whereas DHSs tended to be open in heterochromatin regions under darkness. Extended darkness thereby resulted in more closed chromatin in euchromatic regions and decompaction in heterochromatic regions. We showed that the distribution and proportion of non-TE and TE-related DHSs were quite different. Under extended darkness, DHSs were preferentially located in TE bodies, in contrast to the distribution of the non-TE genes. Bourbousse and colleagues have showed that prolonged exposure to light triggers nuclear growth and the condensation of some heterochromatic regions during *Arabidopsis* postembryonic development, whereas both nuclear growth and low heterochromatin condensation are repressed in the dark^17^. Our results were consistent with light signals controlling nuclear architecture reorganization during *Arabidopsis* seedling establishment, demonstrating that different light conditions influence chromatin accessibility during seedling establishment and in mature plants.

### Association of DHSs with expression regulation of non-TE genes under extended darkness

Through high-resolution mapping of DHSs, we found that extended darkness affected chromatin accessibility. The altered gene expression profiles revealed how plants respond to extended darkness and control condition, thus raising questions concerning whether the chromatin accessibility changes under darkness are associated with differential gene expression. The overlap of differential chromatin accessibility and transcriptional activity of non-TE genes were calculated, and 519 genes with dark-diminished DHSs were identified with down-regulated transcriptional levels under darkness. Photosynthesis and chlorophyll-related genes were significantly enriched among these 519 genes, on the basis of GO term and GSEA, as expected. This finding was consistent with the observation that extended darkness resulted in leaf senescence and lower chlorophyll content compared with control condition. These enriched photosynthesis-related GO terms suggested that under the control condition, the light triggers open chromatin with high DNase I hypersensitivity near genes associated with photosynthesis and chlorophyll and further promotes the expression of these genes. However, under extended darkness, most chromatin regions for non-TE genes tend to be condensed, and the darkness diminishes DHSs near light-responsive genes and represses gene expression. Some other enriched GO terms included leaf morphogenesis and floral organ development, thus potentially explaining the different phenotypes observed between control condition and extended darkness. For example, extended darkness caused stem/petiole elongation and early flowering. Some GO terms, such as “salicylic acid biosynthetic process” and “response to jasmonic acid stimulus” also suggested association between extended darkness and hormone biosynthesis/signal transduction pathways. Therefore, we conclude that extended darkness could trigger chromatin condensation near TSSs of the genes affecting photosynthesis, and leading to changes in plant anatomy and differentially regulated hormone-related genes via the down-regulation of their transcriptional activities, thereafter influencing the *Arabidopsis* growth and development.

Particularly, in the promoter regions of the photosynthesis-related genes, the GLK-binding site “CCAATC” was significantly enriched. GLK (GOLDEN 2-like) is a nuclear-encoded transcription factor and a key regulator in the light-induced transcriptional network. GLK1 is a transcriptional factor in chloroplast retrograde signaling, a process whereby chloroplast components give rise to one or more retrograde signals from the chloroplast to nucleus, to evoke changes in nuclear gene expression that consequently modulate chloroplast function and photosystem^35, 36^. Our results showed that GLK1-induced genes were DHS-diminished and down-regulated, thus leading us to investigate whether chloroplast retrograde signaling was influenced by extended darkness. Through differential expression analysis, we found that many genes located in chloroplasts such as *GUN4, GUN5, SIG2* and *SIG6* were down-regulated or DHS-diminished under extended darkness. These genes were associated with chloroplast biogenesis and transcription in chloroplasts^35, 36^. DHSs located in the GLK1 binding motif were diminished in the promoter regions of photosynthesis-related genes, which potentially explained how repressing GLK1 might also affect the transcriptional regulation of photosynthesis-related genes under extended darkness. This result was consistent with findings from a previously report showing that light influencs on retrograde signaling during photomorphogenesis, In the darkness, *GLK1* expression is also repressed, and the retrograde signaling affects deetiolation^37^. Our results revealed that extended darkness could influences chloroplast retrograde signaling.

### Differential DHSs in TEs and possible association with siRNA changes under extended darkness

By identifying DHSs in different TE families under extended darkness treatment and control condition, we observed a relatively high proportion of DHS-increased TEs under extended darkness, consisting of LTR/Gypsy retrotransposons located in the heterochromatin flanking the centromeres. However the DHSs-diminished TEs under extended darkness were enriched in the Copia, LINE, and MuDR families dispersed throughout the chromosomes. Our results indicated that extended darkness might affect chromatin accessibility in TE regions. Recently, Bourbousse *et al*. have reported the effects of light on chromatin structure by cytogenetics analyses^17^. Their results have shown that the RNA level of *AtGP1*, a Gypsy retrotransposon, is decreased after light exposure. Here, we proposed that extended darkness might activate Gypsy retrotransposons and increase accessibility in heterochromatic regions.

TEs can produce transcripts that are immediately converted into double-stranded RNA and cleaved into siRNA^42^. Through sRNA-seq analysis, we found that changes of TEs in siRNAs were consistent with those of the DHSs. In Gypsy regions, siRNAs were more enriched under extended darkness than under control condition, whereas some siRNAs were more enriched in Copia regions under control condition than under darkness. The consistent changes between siRNA and DHSs indicated that the siRNAs may be derived from TEs, which may be affected by the chromatin structure.

### Extended darkness might affect the RdDM pathway

Recently, transposons have been reported to be activated in response to environmental stress through a combination of loss of RNA-directed DNA methylation (RdDM)^41^. Transcriptional gene silencing (TGS) consists of light-stimulated concurrent processing during seedling development^17^. Bourbousse *et al*. have found that many genes associated with RdDM, such as *DDM1, AGO4* and *AGO6*, have lower RNA levels in darkness than in light^17^. In addition, the leaves of *drd1* and *ddm1* mutants lost less chlorophyll content than wild-type leaves after extended darkness treatment^43^. In the present study, we analysed the changes in the expression and DHS of genes involved in the RdDM pathway under extended darkness. We found that the genes involved in RdDM tended to be down-regulated and/or DHS-diminished under extended darkness treatment (Supplementary Table 10), including *AGO4, DMS3, NRPD1A, CMT3, MET1* and *VIM1*. Thus, we speculated that the RdDM pathway might be influenced by extended darkness. To study changes in DNA methylation under extended darkness, we generated bisulfite-seq (BS-seq) data, but we identified no differences in DNA methylation globally at the whole-genome level between extended darkness and control condition (data not shown), in agreement with the results of Bourbousse^17^.

**Figure 6:**
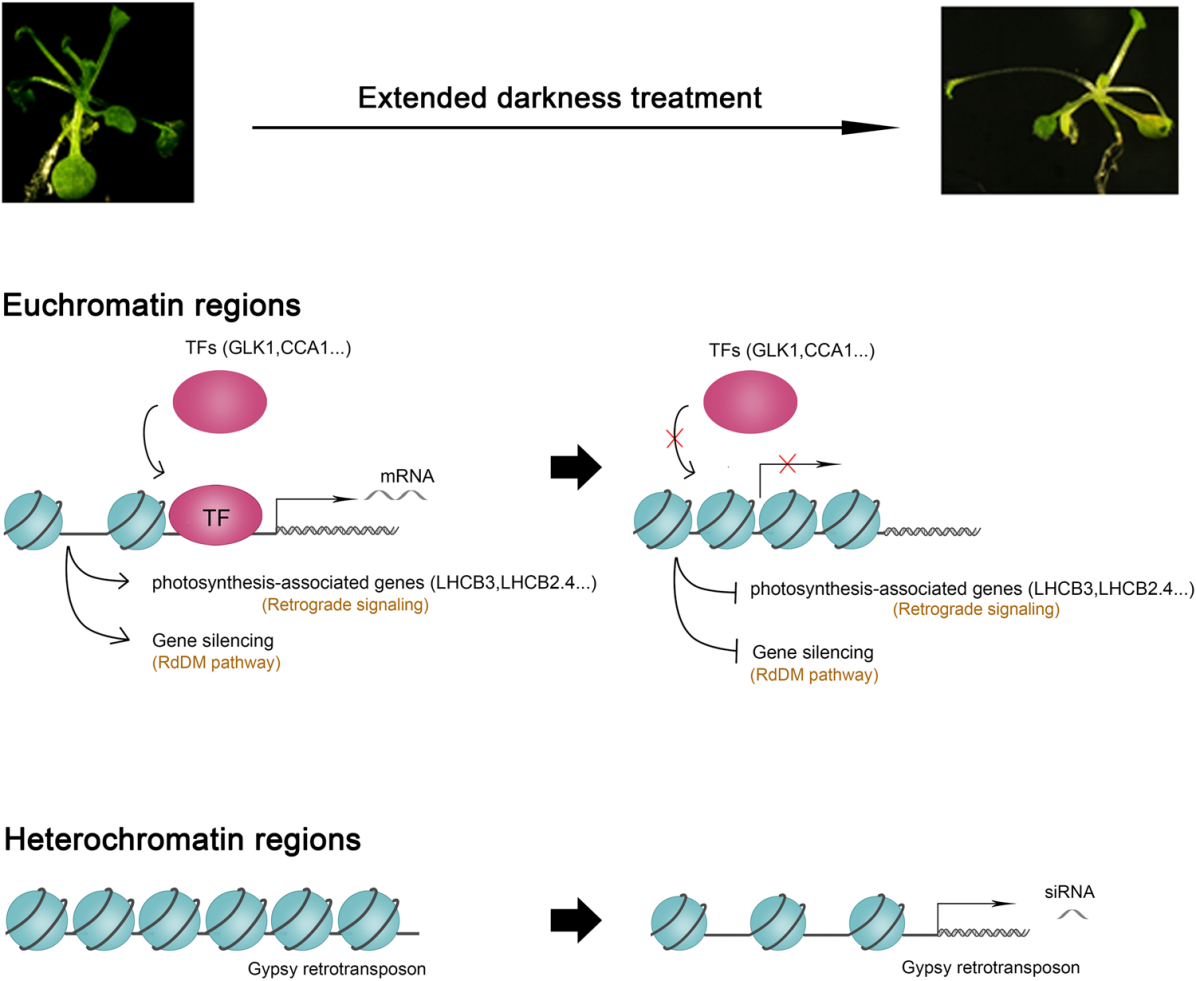
Model for changes in chromatin accessibility during extended darkness. In euchromatin, chromatin becomes closed during extended darkness. This change represses the binding of light-associated TFs, such as GLK1 and CCA1, to DHSs in the promoters of downstream photosynthesis-associated genes. The repression of photosynthesis-associated genes may be influenced by retrograde signaling. In addition, gene silencing, such as the RdDM pathway, is also repressed by closed chromatin. In heterochromatin, chromatis tends to open during extended darkness, and LTR/Gypsy transposons are transcribed into siRNAs.

In summary, we conducted DNase-seq and mRNA-seq to identify differential DHSs and differentially expressed genes, and we revealed chromatin accessibility changes in euchromatic and heterochromatic regions under extended darkness in Arabidopsis (Fig. 6). Our results showed that in non-TE regions, the majority of the DHS-changed genes were DHS-diminished genes under extended darkness. The association analysis of DHSs and expression regulation indicated that light might promote TF binding to DHSs containing conserved motifs within chromatin that is loose and accessible. The modified chromatin structure potentially regulated downstream target gene expression, especially genes involved in photosynthesis and chloroplast retrograde signaling. In TE regions, there was a relatively high proportion of DHS-increased TEs under extended darkness treatment, particularly Gypsy retrotransposons. In addition, the differentially changed DHSs in TE regions might be strongly associated with siRNA changes under extended darkness and control condition. In addition, we found that some genes had increased expression levels but were DHS-diminished during extended darkness treatment. We speculate that other factors are likely to be involved in regulating the extended darkness response, such as miRNA targets and histone modifications. Overall, our findings revealed the changes in chromatin accessibility under extended darkness and might provide answers to fundamental questions about how light signals influence transcription at the genomic scale, but the complicated regulatory mechanism requires further study.

## Materials and Methods

### Plant materials and growth conditions

*Arabidopsis thaliana* (Col-0) seeds were surface-sterilized and sown on half-strength Murashige and Skoog (MS) medium in Petri plates. The seeds were stratified for 3 d at 4 °C and then transferred to a conditioning chamber under 16 h light (22 °C)/8 h dark (19 °C) cycles. *Arabidopsis* plants were grown in Petri plates for 15 days. All the plants subjected to dark treatment in Petri dishes were wrapped in aluminium foil. *Arabidopsis* plants were grown under control condition (see above) as a control. After 3 days, whole plants with and without extended dark treatment were harvested for mRNA-seq, DNase-seq, and sRNA-seq.

### RNA-seq and analysis

Total RNA was extracted using TRIZOL reagent (Invitrogen, CA, USA) and purified using an RNeasy Mini Kit (Qiagen). The RNA samples were extracted from whole *Arabidopsis* whole plants under extended darkness treatment and control condition. RNA-seq libraries were developed from three biological replicates from whole plants under extended darkness treatment and control condition, and sequenced with a HiSeq 2500 Sequencing System (Illumina, San Diego, CA, USA) in the Research Technology Support Facility at Michigan State University.

Sequencing reads of RNA-seq were aligned to the *Arabidopsis* genome (TAIR10) by using TopHat software^44^. Calculation of FPKM (fragments per kilobase of transcript per million mapped reads) and the identification of differentially expressed genes were performed using Cuffdiff in Cufflinks software^32^. Genes showing statistically significant differential expression, on the basis of [FPKM] value > 2-fold change and p-value < 0.05 were identified as differentially expressed genes (DEGs). Gene ontology (GO) enrichment analysis was performed using the agriGO website^30^ and REVIGO^31^.

### DNase-seq and analysis

DNase-seq experiments, including nuclei isolation, DNase I digestion, and DNase-seq library construction, were performed as previously described^25^. DNase-seq libraries were developed from whole plants under extended darkness and normal conditions, and were sequenced by HiSeq 2500 Sequencing System (Illumina, San Diego, CA, USA) in the Research Technology Support Facility at Michigan State University.

Bowtie2 software^45^ was used to align the sequencing reads of DNase-seq to the *Arabidopsis* reference genome (TAIR10) by using default parameters. The peak in different conditions and differentially changed peaks were called by MACS software^46^. The nomodel parameter was set, the d-value parameter was set as 200, and the m-fold was set as 8–30. The CEAS software^29^ was used to analyse the distance between TSSs of genes and the nearest called peaks. We then identified differentially changed DHS peaks located in the region of 3 kb upstream and 1 kb downstream of TSSs as DHS-changed genes. GO enrichment analysis was performed using the agriGO website^30^ and gene set enrichment analysis using plantGSEA^33^.

### Motif analysis

We extracted sequences from the +/− 500 bp regions surrounding the DHS-changed peak summits in 519 DHS-diminished and down-regulated genes under extended darkness as search sequences, and the sequences of 1000 bp regions at upstream and downstream of the DHS-changed peak as background sequences. We analysed the enrichment of motifs from several groups, including the Plant Cis-acting Regulatory DNA Elements (PLACE) database^47^, AthaMap webserver^48^ and PlantCARE database^49^. Significance was determined using an algorithm based on Z score and P-value filtering with a cut-off of 0.05^50^.

### sRNA-seq and analysis

Total RNA was extracted using TRIZOL reagent (Invitrogen) and purified using RNeasy Mini Kits (Qiagen). RNA samples were extracted from *Arabidopsis* whole plants under extended darkness treatment and control condition. Construction of the sRNA libraries and deep-sequencing were carried out by the Beijing Genomics Institute (BGI, Hong Kong, China): isolated total RNA from each sample was separated on 15% denaturing polyacrylamide gels for size selection. Small RNAs (18 to 30 nt) were selected and the developed sRNA libraries were directly sequenced with a HiSeq 2000 Sequencing System (Illumina, San Diego, CA, USA). The sequenced sRNAs were aligned to the genome with SOAP software^51^. These sRNAs were annotated by mapping to sequences including miRNAs, repeats, snRNA, rRNAs, and tRNAs in miRBase, Rfam and GeneBbank. Candidate siRNA reads were identified by mapping to repeat regions. siRNA reads located on TEs were overlapped by using BED Tools software^52^. siRNA-changed TEs were identified by calculating reads per million (RPM) of siRNA for one TE with a cut-off of 1.6 and 0.05 in fold change and P-value, respectively.

## Electronic supplementary material

Supplemental figures and tables

Supplementary Data 1

Supplementary Data 2

Supplementary Data 3

Supplementary Data 4

Supplementary Data 5

Supplementary Data 6

Supplementary Data 7

Supplementary Data 8

Supplementary Data 9

Supplementary Data 10
