# Supplementary material for: Genome-wide mapping of DNase I hypersensitive sites reveals chromatin accessibility changes in Arabidopsis euchromatin and heterochromatin regions under extended darkness
Source: Sci Rep. 2017 Jun 22;7:4093. doi: 10.1038/s41598-017-04524-9 (PMC5481438; doi:10.1038/s41598-017-04524-9)
Supplement: Supplementary file 1 — Supplemental figures and tables [file 41598_2017_4524_MOESM1_ESM.pdf]

Genome-wide mapping of DNase I hypersensitive sites reveals chromatin accessibility changes in *Arabidopsis* euchromatin and heterochromatin regions under extended darkness

Yue Liu[†1], Wenli Zhang[†2], Kang Zhang[1], Qi You[1], Hengyu Yan[1], Yuannian Jiao[3], Jiming Jiang[4], Wenying Xu[1], Zhen Su*[1]

[1]College of Biological Sciences, China Agricultural University, State key Laboratory of Plant Physiology and Biochemistry, Beijing, China, [2]Nanjing Agricultural University, State Key Laboratory for Crop Genetics and Germplasm Enhancement, JCIC-MCP, Nanjing, China, [3]State Key Laboratory of Systematic and Evolutionary Botany, Institute of Botany, The Chinese Academy of Sciences, Beijing, China, [4]University of Wisconsin-Madison, Department of Horticulture, Madison, WI, USA

† These authors contributed equally to this work

* Authors for correspondence

Zhen Su

E-mail: zhensu@cau.edu.cn

Work Phone: +86-10-62731380

Fax: +86-10-62731380

Supplemental figures and tables

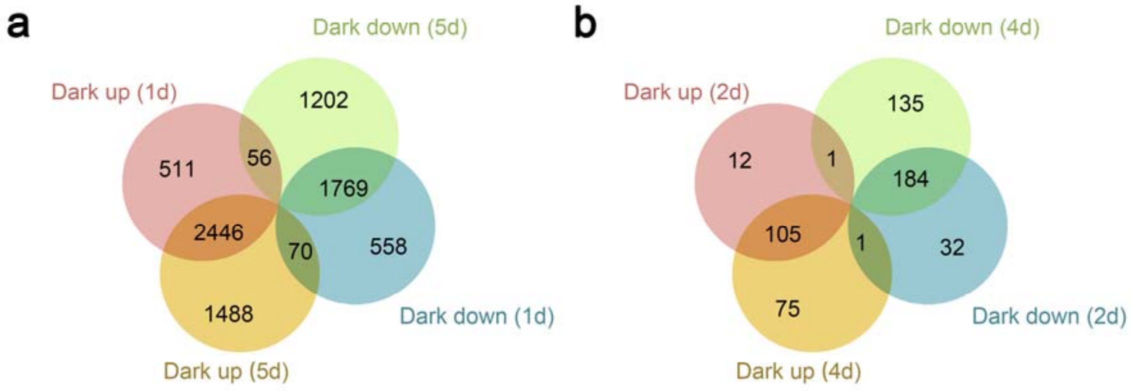

Supplementary Figure 1 | Venn diagrams of gene expression data between different days of extended dark treatment. (**a**) The 1-day and 5-day dark treatment measured by ATH1 GeneChip. (**b**) The 2-day and 4-day dark treatment measured by qRT-PCR.

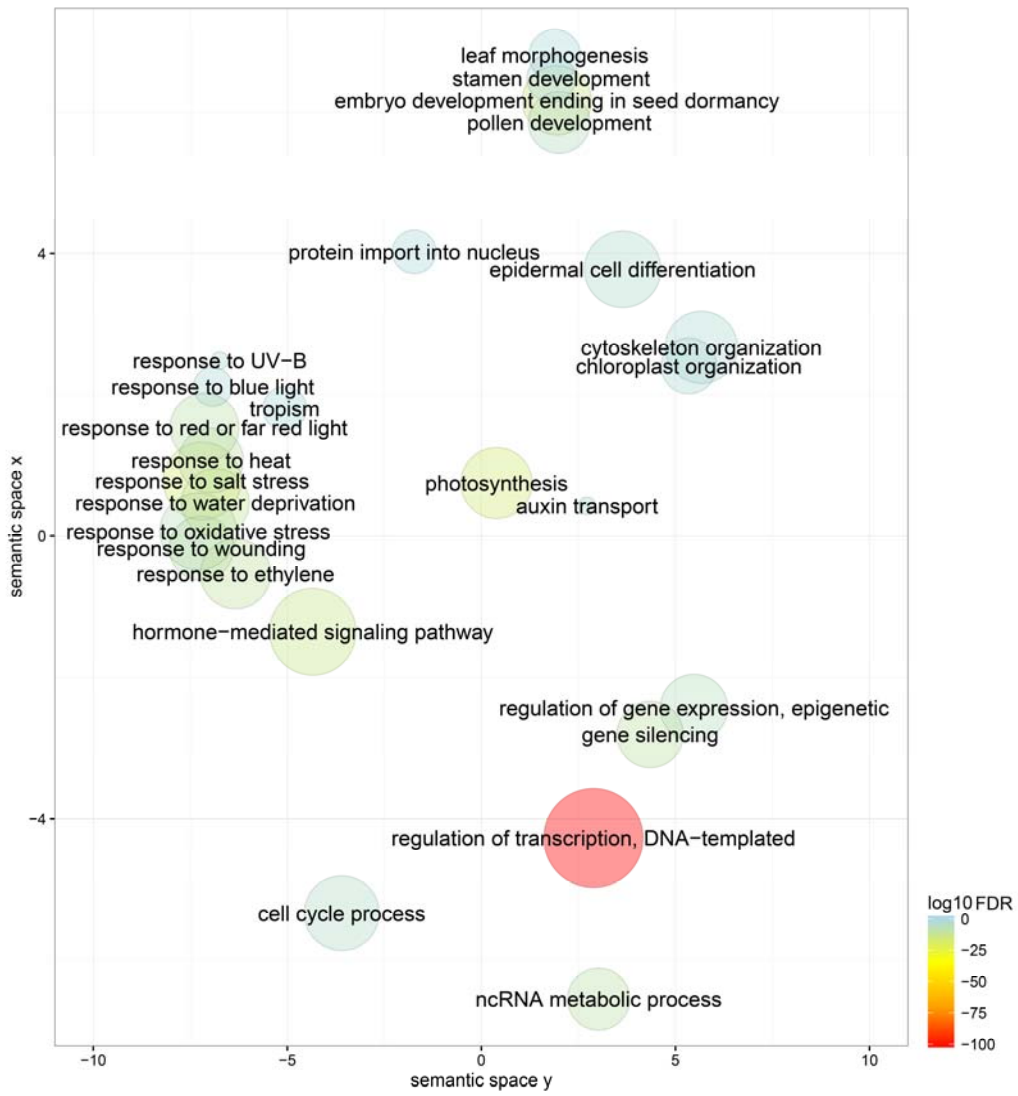

Supplementary Figure 2 | GO enrichment analysis of DHS-diminished genes under extended darkness by agriGO and REVIGO. The scatterplot shows the cluster representatives in a two-dimensional space derived by applying multidimensional scaling to a matrix of significant GO terms with semantic similarities. Bubble color and size indicates the $\log_{10}$(FDR P-value) (legend in bottom right-hand corner).

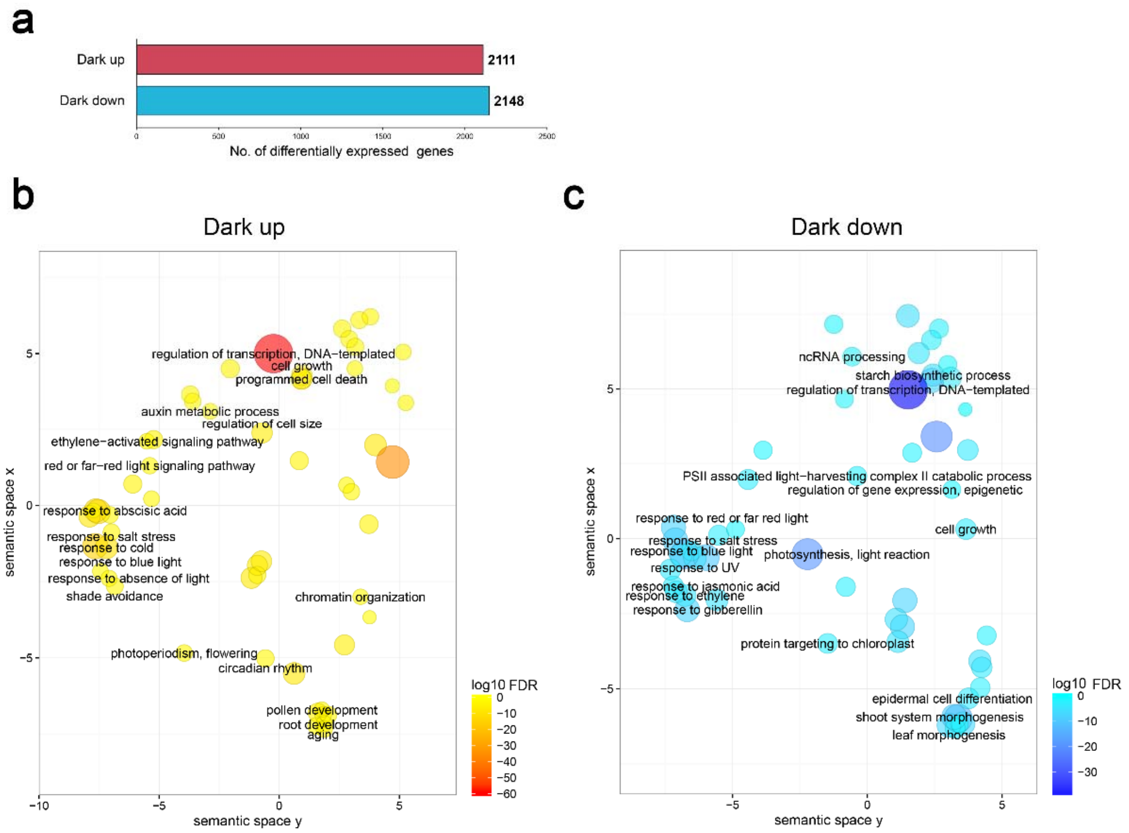

Supplementary Figure 3 | Differentially expressed genes analysis under extended darkness. (**a**) The number of differentially expressed genes under extended darkness. (**b, c**) GO enrichment analysis of up-regulated (b) and down-regulated (c) genes under extended darkness by agriGO and REVIGO. The scatterplot shows the cluster representatives in a two-dimensional space derived by applying multidimensional scaling to a matrix of significant GO terms with semantic similarities. Bubble color and size indicates the $\log_{10}$(FDR P-value) (legend in the bottom right-hand corner).

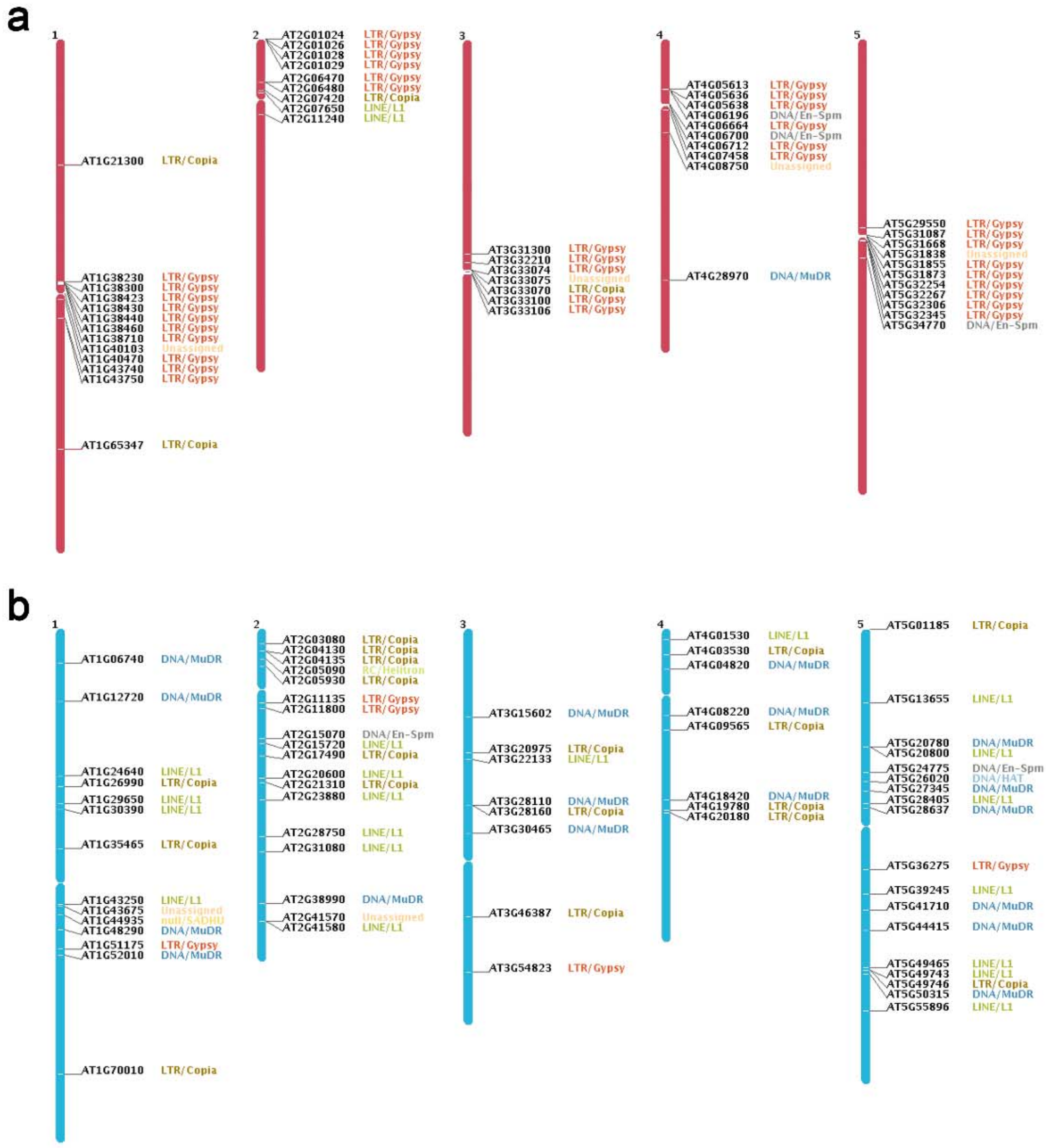

Supplementary Figure 4 | Locations of DHS-changed TEs on chromosomes. (**a**) DHS-increased TEs under extended darkness. (**b**) DHS-diminished TEs under extended darkness.

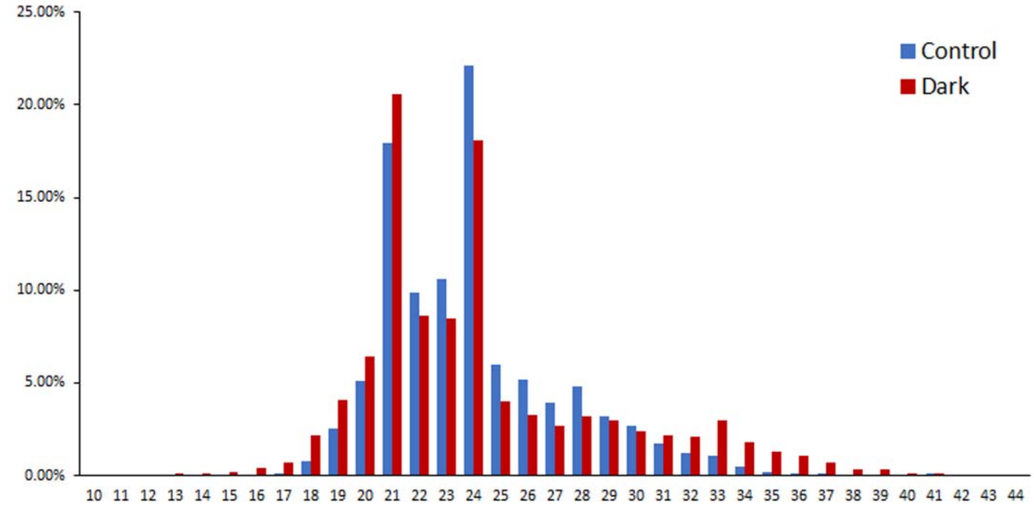

Supplementary Figure 5 | Length distribution of sequenced small RNAs.

Supplementary Table 1 | Statistical summary of DNase I sequencing libraries and identified peak number.

Sample	Total reads	Mapped Reads	Mapping rate (%)	Called peaks
Dark	91,141,444	89,146,524	97.81	5,948
Control	88,954,436	82,794,608	93.08	10,380

Supplementary Table 2 | Selected enriched GO terms among DHS-diminished genes under extended darkness.

GO term	GO name	Qnum	B/Rnum	FDR
GO:0045449	regulation of transcription	209	231	1.10E-104
GO:0006412	translation	76	143	2.50E-26
GO:0009651	response to salt stress	44	70	3.40E-17
GO:0015979	photosynthesis	34	39	2.90E-16
GO:0044265	cellular macromolecule catabolic process	34	42	1.40E-15
GO:0009793	embryonic development ending in seed dormancy	40	72	2.60E-14
GO:0034637	cellular carbohydrate biosynthetic process	28	31	1.10E-13
GO:0009755	hormone-mediated signaling pathway	35	57	1.70E-13
GO:0046483	heterocycle metabolic process	43	93	2.90E-13
GO:0042742	defense response to bacterium	25	26	1.20E-12
GO:0009414	response to water deprivation	26	32	5.30E-12
GO:0006457	protein folding	26	36	3.10E-11
GO:0048589	developmental growth	21	21	6.90E-11
GO:0009723	response to ethylene stimulus	23	30	2.50E-10
GO:0006732	coenzyme metabolic process	21	25	6.00E-10
GO:0009611	response to wounding	22	30	1.30E-09
GO:0016458	gene silencing	21	27	1.60E-09
GO:0009639	response to red or far red light	34	83	1.80E-09
GO:0051188	cofactor biosynthetic process	22	31	1.90E-09
GO:0009408	response to heat	18	18	2.40E-09
GO:0034660	ncRNA metabolic process	22	32	2.90E-09
GO:0008652	cellular amino acid biosynthetic process	20	26	4.80E-09
GO:0044264	cellular polysaccharide metabolic process	17	19	2.20E-08
GO:0032989	cellular component morphogenesis	19	28	5.70E-08
GO:0010200	response to chitin	24	52	1.50E-07
GO:0006979	response to oxidative stress	31	94	6.30E-07
GO:0006399	tRNA metabolic process	13	13	8.20E-07
GO:0006091	generation of precursor metabolites and energy	22	49	8.90E-07
GO:0048468	cell development	14	17	1.20E-06
GO:0040029	regulation of gene expression, epigenetic	20	41	1.30E-06
GO:0009555	pollen development	18	35	3.10E-06
GO:0006633	fatty acid biosynthetic process	12	13	4.70E-06
GO:0048522	positive regulation of cellular process	16	28	5.10E-06

GO:0043623	cellular protein complex assembly	14	21	7.30E-06
GO:0009117	nucleotide metabolic process	16	30	1.00E-05
GO:0016049	cell growth	17	35	1.20E-05
GO:0010608	posttranscriptional regulation of gene expression	15	27	1.50E-05
GO:0009699	phenylpropanoid biosynthetic process	16	33	2.50E-05
GO:0016192	vesicle-mediated transport	23	69	2.80E-05
GO:0022402	cell cycle process	14	26	4.50E-05
GO:0042545	cell wall modification	14	26	4.50E-05
GO:0006790	sulfur metabolic process	16	39	0.00014
GO:0048878	chemical homeostasis	10	14	0.00021
GO:0007389	pattern specification process	10	15	0.00032
GO:0006855	multidrug transport	8	8	0.00032
GO:0016052	carbohydrate catabolic process	12	26	0.00073
GO:0043603	cellular amide metabolic process	7	7	0.0011
GO:0007010	cytoskeleton organization	13	33	0.0013
GO:0009165	nucleotide biosynthetic process	11	24	0.0016
GO:0009886	post-embryonic morphogenesis	12	29	0.0016
GO:0010224	response to UV-B	7	8	0.0018
GO:0048581	negative regulation of post-embryonic development	7	8	0.0018
GO:0030001	metal ion transport	23	98	0.0027
GO:0000154	rRNA modification	7	9	0.003
GO:0009913	epidermal cell differentiation	10	22	0.0033
GO:0006281	DNA repair	17	61	0.0035
GO:0019362	pyridine nucleotide metabolic process	6	6	0.0035
GO:0009637	response to blue light	15	49	0.0035
GO:0010876	lipid localization	9	18	0.0039
GO:0019318	hexose metabolic process	12	34	0.0051
GO:0042401	cellular biogenic amine biosynthetic process	6	7	0.0059
GO:0045454	cell redox homeostasis	6	7	0.0059
GO:0048285	organelle fission	6	8	0.0095
GO:0000278	mitotic cell cycle	5	5	0.012
GO:0048443	stamen development	5	5	0.012
GO:0009808	lignin metabolic process	6	9	0.014
GO:0009658	chloroplast organization	6	9	0.014
GO:0040034	regulation of development, heterochronic	6	9	0.014
GO:0048609	reproductive process in a multicellular organism	6	9	0.014
GO:0045087	innate immune response	42	266	0.017
GO:0009225	nucleotide-sugar metabolic process	5	6	0.019
GO:0009629	response to gravity	5	6	0.019

GO:0009965	leaf morphogenesis	7	15	0.025
GO:0016570	histone modification	5	7	0.03
GO:0009606	tropism	5	7	0.03
GO:0034220	ion transmembrane transport	5	7	0.03
GO:0006606	protein import into nucleus	5	7	0.03
GO:0015992	proton transport	5	7	0.03
GO:0006820	anion transport	5	7	0.03
GO:0046246	terpene biosynthetic process	5	8	0.043
GO:0009850	auxin metabolic process	5	8	0.043
GO:0009938	negative regulation of gibberellic acid mediated signaling pathway	5	8	0.043
GO:0010014	meristem initiation	5	8	0.043
GO:0060918	auxin transport	5	8	0.043
GO:0032879	regulation of localization	5	8	0.043

Supplementary Table 3 | Statistical summary of RNA sequencing libraries.

Sample	Total reads	Mapped Reads	Mapping rate (%)	Properly paired (%)
Dark rep1	75,611,298	70,097,103	92.70	89.00
Dark rep2	26,292,540	25,718,503	97.80	96.00
Dark rep3	40,605,160	38,806,040	95.60	92.60
Control rep1	48,280,292	45,077,989	93.40	90.30
Control rep2	28,919,402	28,228,112	97.60	95.80
Control rep3	42,309,538	40,862,281	96.60	94.40

Supplementary Table 4 | Selected enriched GO terms among down-regulated genes under extended darkness.

GO term	GO name	Qnum	B/Rnum	FDR
GO:0045449	regulation of transcription	101	231	1.50E-40
GO:0006631	fatty acid metabolic process	35	40	5.60E-21
GO:0019684	photosynthesis, light reaction	43	78	3.90E-20
GO:0009611	response to wounding	26	30	1.60E-15
GO:0009793	embryonic development ending in seed dormancy	34	72	2.00E-14
GO:0042742	defense response to bacterium	19	26	1.60E-10
GO:0010016	shoot morphogenesis	18	27	1.70E-09
GO:0009651	response to salt stress	26	70	2.80E-09
GO:0009739	response to gibberellin stimulus	15	19	1.20E-08
GO:0009639	response to red or far red light	27	83	1.30E-08
GO:0009657	plastid organization	28	91	1.80E-08
GO:0009165	nucleotide biosynthetic process	16	24	1.80E-08
GO:0043623	cellular protein complex assembly	15	21	2.90E-08
GO:0009642	response to light intensity	24	70	4.80E-08
GO:0006720	isoprenoid metabolic process	18	36	4.80E-08
GO:0009965	leaf morphogenesis	13	15	7.30E-08
GO:0048507	meristem development	14	22	2.90E-07
GO:0006779	porphyrin biosynthetic process	18	48	1.50E-06
GO:0009411	response to UV	20	61	1.70E-06
GO:0009414	response to water deprivation	15	32	1.80E-06
GO:0048437	floral organ development	13	24	3.60E-06
GO:0042254	ribosome biogenesis	13	25	5.10E-06
GO:0019252	starch biosynthetic process	9	9	8.00E-06
GO:0048468	cell development	11	17	8.40E-06
GO:0009624	response to nematode	18	59	1.70E-05
GO:0009723	response to ethylene stimulus	13	30	2.40E-05
GO:0009743	response to carbohydrate stimulus	18	62	3.00E-05
GO:0048285	organelle fission	8	8	3.40E-05
GO:0009809	lignin biosynthetic process	8	9	6.00E-05
GO:0009813	flavonoid biosynthetic process	17	61	8.50E-05
GO:0048438	floral whorl development	11	24	9.50E-05
GO:0042542	response to hydrogen peroxide	13	38	1.70E-04
GO:0009913	epidermal cell differentiation	10	22	2.50E-04
GO:0043455	regulation of secondary metabolic process	7	8	2.50E-04
GO:0016049	cell growth	12	35	3.50E-04
GO:0040034	regulation of development, heterochronic	7	9	4.00E-04
GO:0010374	stomatal complex development	6	6	5.90E-04

GO:0019253	reductive pentose-phosphate cycle	6	6	5.90E-04
GO:0019362	pyridine nucleotide metabolic process	6	6	5.90E-04
GO:0009753	response to jasmonic acid stimulus	26	152	6.50E-04
GO:0016036	cellular response to phosphate starvation	6	7	9.90E-04
GO:0010205	photoinhibition	6	7	9.90E-04
GO:0016570	histone modification	6	7	9.90E-04
GO:0019761	glucosinolate biosynthetic process	11	34	1.10E-03
GO:0009637	response to blue light	13	49	1.30E-03
GO:0009886	post-embryonic morphogenesis	10	29	1.40E-03
GO:0045036	protein targeting to chloroplast	6	8	1.60E-03
GO:0045165	cell fate commitment	5	5	2.40E-03
GO:0009082	branched chain family amino acid biosynthetic process	5	5	2.40E-03
GO:0010304	PSII associated light-harvesting complex II catabolic process	5	6	4.20E-03
GO:0006857	oligopeptide transport	14	66	5.10E-03
GO:0045595	regulation of cell differentiation	5	7	6.60E-03
GO:0042371	vitamin K biosynthetic process	5	7	6.60E-03
GO:0016122	xanthophyll metabolic process	6	12	7.10E-03
GO:0005983	starch catabolic process	6	13	9.60E-03
GO:0034470	ncRNA processing	8	26	1.10E-02
GO:0042538	hyperosmotic salinity response	5	9	1.40E-02
GO:0019288	isopentenyl diphosphate biosynthetic process, mevalonate-independent pathway	5	9	1.40E-02
GO:0006325	chromatin organization	7	21	1.60E-02
GO:0006268	DNA unwinding during replication	5	10	1.90E-02
GO:0006281	DNA repair	12	61	2.00E-02
GO:0009695	jasmonic acid biosynthetic process	7	24	2.80E-02
GO:0006007	glucose catabolic process	12	65	3.10E-02
GO:0048580	regulation of post-embryonic development	8	32	3.20E-02
GO:0010228	vegetative to reproductive phase transition of meristem	13	75	3.40E-02
GO:0009862	systemic acquired resistance, salicylic acid mediated signaling pathway	5	12	3.40E-02
GO:0009620	response to fungus	18	124	3.40E-02
GO:0006270	DNA replication initiation	5	12	3.40E-02
GO:0040029	regulation of gene expression, epigenetic	9	41	3.70E-02

Supplementary Table 5 | Selected enriched GO terms among up-regulated genes under extended darkness.

GO term	GO name	Qnum	B/Rnum	FDR
GO:0045449	regulation of transcription	127	231	8.00E-61
GO:0019219	regulation of nucleobase, nucleoside, nucleotide and nucleic acid metabolic process	128	239	2.40E-60
GO:0010468	regulation of gene expression	134	275	1.50E-59
GO:0006464	protein modification process	78	144	6.90E-37
GO:0009651	response to salt stress	35	70	1.20E-15
GO:0010200	response to chitin	28	52	4.20E-13
GO:0009723	response to ethylene stimulus	21	30	2.10E-11
GO:0009409	response to cold	19	28	3.90E-10
GO:0048580	regulation of post-embryonic development	18	32	1.10E-08
GO:0044265	cellular macromolecule catabolic process	20	42	1.10E-08
GO:0010038	response to metal ion	15	21	2.70E-08
GO:0016192	vesicle-mediated transport	23	69	1.20E-07
GO:0006886	intracellular protein transport	19	50	4.70E-07
GO:0012501	programmed cell death	13	20	7.00E-07
GO:0008361	regulation of cell size	16	35	7.80E-07
GO:0016049	cell growth	16	35	7.80E-07
GO:0010876	lipid localization	12	18	1.80E-06
GO:0032989	cellular component morphogenesis	14	28	2.20E-06
GO:0009414	response to water deprivation	13	32	3.80E-05
GO:0009793	embryonic development ending in seed dormancy	19	72	4.10E-05
GO:0006457	protein folding	13	36	1.00E-04
GO:0045454	cell redox homeostasis	7	7	1.50E-04
GO:0048583	regulation of response to stimulus	13	40	2.50E-04
GO:0010118	stomatal movement	7	8	2.50E-04
GO:0007166	cell surface receptor linked signaling pathway	9	17	2.60E-04
GO:0006631	fatty acid metabolic process	12	40	9.60E-04
GO:0007623	circadian rhythm	13	47	9.60E-04
GO:0048523	negative regulation of cellular process	13	47	9.60E-04
GO:0046395	carboxylic acid catabolic process	16	73	1.50E-03
GO:0030001	metal ion transport	19	98	1.50E-03
GO:0046394	carboxylic acid biosynthetic process	14	58	1.60E-03
GO:0006575	cellular amino acid derivative metabolic process	15	66	1.70E-03
GO:0009408	response to heat	8	18	1.80E-03

GO:0048364	root development	11	37	1.90E-03
GO:0008610	lipid biosynthetic process	18	94	2.30E-03
GO:0009641	shade avoidance	5	5	2.60E-03
GO:0048878	chemical homeostasis	7	14	2.70E-03
GO:0042742	defense response to bacterium	9	26	3.00E-03
GO:0009908	flower development	16	80	3.30E-03
GO:0007389	pattern specification process	7	15	3.60E-03
GO:0007568	aging	15	73	4.00E-03
GO:0033554	cellular response to stress	17	91	4.20E-03
GO:0048532	anatomical structure arrangement	5	6	4.30E-03
GO:0009081	branched chain family amino acid metabolic process	5	6	4.30E-03
GO:0048573	photoperiodism, flowering	5	6	4.30E-03
GO:0048522	positive regulation of cellular process	9	28	4.40E-03
GO:0006914	autophagy	8	22	4.80E-03
GO:0010017	red or far-red light signaling pathway	9	29	5.40E-03
GO:0009887	organ morphogenesis	8	23	6.10E-03
GO:0009646	response to absence of light	6	12	7.10E-03
GO:0016138	glycoside biosynthetic process	5	8	9.50E-03
GO:0009850	auxin metabolic process	5	8	9.50E-03
GO:0006855	multidrug transport	5	8	9.50E-03
GO:0009914	hormone transport	5	8	9.50E-03
GO:0045935	positive regulation of nucleobase, nucleoside, nucleotide and nucleic acid metabolic process	5	8	9.50E-03
GO:0044264	cellular polysaccharide metabolic process	7	19	9.60E-03
GO:0009637	response to blue light	11	49	1.20E-02
GO:0009873	ethylene mediated signaling pathway	12	58	1.30E-02
GO:0019438	aromatic compound biosynthetic process	11	50	1.30E-02
GO:0005984	disaccharide metabolic process	5	9	1.40E-02
GO:0044242	cellular lipid catabolic process	12	59	1.40E-02
GO:0010604	positive regulation of macromolecule metabolic process	5	9	1.40E-02
GO:0009555	pollen development	9	35	1.50E-02
GO:0006325	chromatin organization	7	21	1.50E-02
GO:0048468	cell development	6	17	2.60E-02
GO:0009737	response to abscisic acid stimulus	32	276	2.70E-02
GO:0044271	cellular nitrogen compound biosynthetic process	13	76	3.30E-02
GO:0042545	cell wall modification	7	26	3.90E-02
GO:0010016	shoot morphogenesis	7	27	4.60E-02

Supplementary Table 6 | Selected enriched GO terms among DHS-diminished and down-regulated genes under extended darkness.

GO term	GO name	Qnum	B/Rnum	FDR
GO:0015979	photosynthesis	25	39	1.50E-26
GO:0045449	regulation of transcription	33	231	3.20E-18
GO:0006952	defense response	23	82	3.40E-18
GO:0010038	response to metal ion	11	21	3.70E-11
GO:0016070	RNA metabolic process	24	232	1.10E-10
GO:0009409	response to cold	9	28	8.70E-08
GO:0009733	response to auxin stimulus	9	29	1.10E-07
GO:0006733	oxidoreduction coenzyme metabolic process	6	6	2.20E-07
GO:0009639	response to red or far red light	12	83	5.90E-07
GO:0016138	glycoside biosynthetic process	6	8	6.40E-07
GO:0009408	response to heat	7	18	1.30E-06
GO:0012501	programmed cell death	7	20	2.30E-06
GO:0005982	starch metabolic process	8	33	3.00E-06
GO:0009767	photosynthetic electron transport chain	5	5	3.00E-06
GO:0009637	response to blue light	9	49	4.00E-06
GO:0008652	cellular amino acid biosynthetic process	7	26	9.00E-06
GO:0009751	response to salicylic acid stimulus	5	7	9.00E-06
GO:0055085	transmembrane transport	8	44	1.80E-05
GO:0009611	response to wounding	7	30	1.90E-05
GO:0043623	cellular protein complex assembly	6	21	3.80E-05
GO:0009653	anatomical structure morphogenesis	10	90	4.80E-05
GO:0006979	response to oxidative stress	10	94	6.70E-05
GO:0070887	cellular response to chemical stimulus	8	62	0.00015
GO:0046148	pigment biosynthetic process	9	83	0.00016
GO:0006259	DNA metabolic process	9	92	0.00032
GO:0006412	translation	11	143	0.00035
GO:0009814	defense response, incompatible interaction	9	95	0.0004
GO:0048437	floral organ development	5	24	0.00077
GO:0030154	cell differentiation	6	43	0.0011
GO:0055086	nucleobase, nucleoside and nucleotide metabolic process	6	46	0.0015
GO:0065009	regulation of molecular function	5	32	0.0024
GO:0040007	growth	6	51	0.0024
GO:0019318	hexose metabolic process	5	34	0.0031
GO:0006457	protein folding	5	36	0.0039

GO:0048519	negative regulation of biological process	7	84	0.0052
GO:0032787	monocarboxylic acid metabolic process	7	85	0.0055
GO:0009813	flavonoid biosynthetic process	6	61	0.0056
GO:0050832	defense response to fungus	7	86	0.0058
GO:0033554	cellular response to stress	7	91	0.0078
GO:0009108	coenzyme biosynthetic process	6	69	0.0098
GO:0009250	glucan biosynthetic process	5	49	0.013
GO:0048229	gametophyte development	5	52	0.016
GO:0010200	response to chitin	5	52	0.016
GO:0009110	vitamin biosynthetic process	5	58	0.024
GO:0018130	heterocycle biosynthetic process	6	86	0.026
GO:0009411	response to UV	5	61	0.03
GO:0009066	aspartate family amino acid metabolic process	5	62	0.031
GO:0008610	lipid biosynthetic process	6	94	0.038
GO:0006857	oligopeptide transport	5	66	0.039
GO:0044275	cellular carbohydrate catabolic process	6	98	0.046
GO:0009642	response to light intensity	5	70	0.049

Supplementary Table 7 | Selected enriched GO terms among DHS-diminished and up-regulated genes under extended darkness.

GO term	GO name	Qnum	B/Rnum	FDR
GO:0045449	regulation of transcription	17	231	2.30E-12
GO:0010033	response to organic substance	14	210	3.80E-10
GO:0043687	post-translational protein modification	7	36	4.20E-08
GO:0009409	response to cold	5	28	9.70E-06
GO:0016070	RNA metabolic process	8	232	0.00055
GO:0048519	negative regulation of biological process	5	84	0.0011
GO:0006810	transport	9	540	0.028

Supplementary Table 8 | Enriched motifs in DHSs around photosynthesis-related genes in DHS-diminished and down-regulated genes under extended darkness.

Accession	Name	Sequence	Count	Z-score	P-value
plantCARE000024	A_box	TATCCATCCATCC	38	4.76	1.00E-06
plantCARE000309	Prolamin_box	TGACATGTAAAGTTAAT	56	4.57	2.40E-06
plantCARE000364	TATA_box	TATATAAAGG	81	4.36	6.60E-06
plantCARE000263	LAMP_element	CCTTATCCA	103	4.29	8.90E-06
S000105	RYREPEATGMGY2	CATGCAT	103	4.29	8.90E-06
plantCARE000007	4cl_CMA1b	ATTCCGATAAACT	72	3.97	3.61E-05
plantCARE000191	G_box	GCCACGTGTCACTC	27	3.79	7.43E-05
S000282	CE3OSOSEM	AACGCGTGTC	18	3.77	8.30E-05
plantCARE000026	ABRE	ACGTACGTGTCGCGC	19	3.73	9.45E-05
plantCARE000328	Sbp_CMA1a	CTCTATCTTCTCCGG	19	3.59	1.67E-04
S000144	EBOXBNNAPA	CANNTG	255	3.42	3.17E-04
S000118	ABRE3HVA22	GCCACGTACA	52	3.32	4.46E-04
plantCARE000368	TATA_box	TATATGT	61	3.27	5.33E-04
S000292	DPBFCOREDCDC3	ACACNNG	84	3.17	7.59E-04
plantCARE000260	JERE	AGACCGCC	84	3.17	7.59E-04
plantCARE000139	Chs_unit_1_m2	TCCGGTGGCCGTCCCTC	14	3.16	7.84E-04
S000302	LREBOXIPCCHS1	AACCTAACCT	73	3.13	8.74E-04
S000156	SARECAMV	CTGACGTAAGGGATGACGCAC	73	3.13	8.74E-04
plantCARE000059	ATC_motif	AGTAATCT	18	3.08	1.02E-03
S000204	BOX2PSGS2	TCTAAGCAAAG	13	2.98	1.42E-03
plantCARE000314	Prolamin_box	TGCCATGTAAAGATGAC	7	2.98	1.45E-03
plantCARE000060	ATC_motif	GCCAATCC	7	2.98	1.45E-03
S000127	RBCSCONSENSUS	AATCCAA	43	2.96	1.53E-03
S000142	ELRECOREPCRP1	TTGACC	23	2.87	2.05E-03
plantCARE000157	Footprint_A	AGTACATCTCATCCATTT	9	2.79	2.68E-03
S000131	ACGTCBOX	GACGTC	22	2.63	4.26E-03
plantCARE000111	CCAAT_box	CAACGG	30	2.60	4.71E-03
plantCARE000049	AE_box	AGAAACWT	181	2.59	4.77E-03
plantCARE000369	TATA_box	TATATTTATATTT	5	2.58	4.90E-03
S000180	MYBST1	GGATA	306	2.56	5.18E-03
S000014	ABRECE1HVA22	TGCCACCGG	306	2.56	5.18E-03
S000317	ATHB1ATCONSENSUS	CAATWATTG	5	2.56	5.25E-03
plantCARE000237	HSE	GAAGAAAATGTTTTAAAAACT	14	2.56	5.28E-03
plantCARE000399	Unnamed_1	TCTTACACGTGGCACCTC	5	2.50	6.23E-03
S000267	OCETYPEINTHISTONE	CCACGTCANCGATCCGCG	972	2.45	7.08E-03
S000494	EECCRCAH1	GANTTNC	154	2.40	8.10E-03
S000174	MYCATRD22	CACATG	18	2.39	8.46E-03
plantCARE000388	TGGCA	GACACCAAGTGGCA	38	2.35	9.40E-03
plantCARE000235	HSE	CTAGAACTGTAGAGAAATCTCCAGAACTTACCAGAAGCTACA	43	2.33	1.00E-02
S000183	SP8BFIBSP8AIB	ACTGTGTA	17	2.32	1.02E-02

plantCARE000013	4cl_CMA2b	TCTCACCAACCMCA	17	2.32	1.03E-02
S000219	ABREBZMRAB28	TCCACGTCTC	28	2.28	1.13E-02
S000085	INTRONUPPER	MAGGTAAGT	8	2.27	1.15E-02
plantCARE000032	ACA_motif	AATCACAACCATA	36	2.27	1.16E-02
S000116	OCTAMOTIF2	CGCGGCAT	94	2.25	1.21E-02
**GLK binding motif**	**GLK binding motif**	**CCAATC**	**59**	**2.22**	**1.33E-02**
S000348	CARG2ATAP3	CTTACCTTTCATGGATTA	5	2.21	1.37E-02
S000356	MREATCHS	TCTAACCTACCA	5	2.14	1.63E-02
plantCARE000286	MSA_like	TGCAACGGC	58	2.13	1.66E-02
S000330	D3GMAUX28	TATTTGCTTAA	47	2.09	1.83E-02
plantCARE000193	G_box	GCCTTGTGTAG	3	2.07	1.90E-02
S000324	GBOXPC	ACCACGTGGC	3	2.07	1.90E-02
S000264	RYREPEATBNNAPA	CATGCA	9	2.07	1.93E-02
plantCARE000422	Unnamed_6	TATAAATATCT	26	2.07	1.94E-02
plantCARE000174	GATT_motif	CTCCTGATTGGA	11	2.05	2.03E-02
plantCARE000420	Unnamed_6	ATCCACCTTTCGTGTACC	45	2.04	2.09E-02
plantCARE000383	TC_rich_repeats	ATTCTCTAAC	238	1.97	2.43E-02
plantCARE000054	As_2_box	GATAATGATG	28	1.95	2.55E-02
S000088	POLASIG3	AATAAT	308	1.89	2.91E-02
plantCARE000117	CGT_motif	CCTGAACGC	308	1.89	2.91E-02
S000019	ACGTSEED3	GTACGTGGCG	67	1.88	3.00E-02
S000159	TCA1MOTIF	TCATCTTCTT	3	1.88	3.01E-02
S000430	GCCCORE	GCCGCC	10	1.88	3.03E-02
S000250	LTRE1HVBLT49	CCGAAA	140	1.85	3.18E-02
S000048	GLUTEBP1OS	AAGCAACACACAAC	51	1.83	3.38E-02
S000307	ELRE2PCPAL1	ATTCTCACCTACCA	141	1.82	3.41E-02
plantCARE000316	Prolamin_box	TGTAGTGTAAAGTGAAA	464	1.81	3.49E-02
plantCARE000253	I_box	GATAKGG	50	1.81	3.51E-02
S000483	SORLIP2AT	GGGCC	4	1.80	3.62E-02
plantCARE000100	CAAT_box	CTCCAATAG	49	1.78	3.74E-02
plantCARE000267	L_box	CTACCTACCCTCTAATATC	2	1.75	3.99E-02
S000507	ABRERATCAL	MACGYGB	2	1.73	4.14E-02
S000393	ARECOREZMGAPC4	AGCAACGGTC	8	1.72	4.28E-02
S000242	NRRBNEXTA	TAGTGGAT	2	1.69	4.52E-02
S000133	ABREZMRAB28	CCACGTGG	2	1.69	4.52E-02
S000203	TATABOX5	TTATTT	2	1.67	4.70E-02
plantCARE000161	GAG_motif	RGAGATG	2	1.67	4.75E-02
plantCARE000092	Box_W2	TTCAGCCATCAAAGTTGACC	2	1.65	4.90E-02

Supplementary Table 9 | Changes in genes involved in the retrograde pathway and interacting components under extended darkness in terms of expression and DHS.

Retrograde Pathway / Interacting Components	Gene Symbol	Locus ID	RNA-seq	DHS-changed significantly	DHS peak only in one condition
Tetrapyrroles	GUN1	AT2G31400			
	GUN2	AT2G26670			
	GUN3	AT3G09150			
	GUN4	AT3G59400	down-regulated	DHS-diminished	Only in Control
	GUN5 /CHLH	AT5G13630	down-regulated	DHS-diminished	Only in Control
	GUN6 /FC1	AT5G26030		DHS-diminished	Only in Control
	HSP90.1 / HSP81-1	AT5G52640	down-regulated		
	HSP81-2	AT5G56030	down-regulated		Only in Control
	HSP90.3 / HSP81-3	AT5G56010	down-regulated		
	HSP90.5 / CR88	AT2G04030	down-regulated		Only in Control
	HY5	AT5G11260			Only in Control
	CRY1	AT4G08920			Only in Control
	PAPP5	AT2G42810			
	ABI4	AT2G40220			
	PTM	AT5G35210			Only in Control
	LHCb1.2	AT1G29910		DHS-diminished	Only in Control
Plastid Transcription and Translation	SIG2	AT1G08540	down-regulated		Only in Control
	SIG6	AT2G36990	down-regulated		Only in Control
	PRIN2	AT1G10522			
	GLK1	AT2G20570	down-regulated		
	GLK2	AT5G44190			
	SG1	AT3G18420			Only in Control
Singlet oxygen ($^{1}O_2$)	CH1 / CAO	AT1G44446	down-regulated	DHS-diminished	Only in Control
	FLU	AT3G14110			
	EX1	AT4G33630		DHS-diminished	Only in Control
	EX2	AT1G27510			
	MBS	AV621044B			
	MBS1	AT3G02790			Only in Control
	MBS2	AT5G16470			
	AAA-ATPase	AT3G28580		DHS-diminished	Only in Control
Photosynthetic Redox State	STN7	AT1G68830	down-regulated		
	PRORS1-1	AT5G52520	down-regulated	DHS-diminished	Only in Control
	STZ / ZAT10	AT1G27730			
	Rap2.4a	AT1G22190	up-regulated		Only in Control
	HSFA1D	AT1G32330			

	HSFA2	AT2G26150	down-regulated	DHS-diminished	Only in Control
	HSFA3	AT5G03720	up-regulated		
	CDKE1	AT5G63610			
SAL1-PAP	SAL1	AT5G63980			
	XRN2	AT5G42540			Only in Control
	XRN3	AT1G75660			
	XRN4 / EIN5	AT1G54490			
	APX2	AT3G09640			

Supplementary Table 10 | Changes in genes associated with RdDM under extended darkness and control condition.

Gene Name	Locus ID	DNase-seq			RNA-seq				
		Control	Dark	Changes	Control-FPKM	Dark-FPKM	log2(Control/ Dark)	P-value	Changes
AGO1	AT1G48410	yes	no		54.20	47.88	0.18	0.80	
AGO4	AT2G27040	yes	no		15.71	6.89	1.19	0.05	
AGO6	AT2G32940	yes	yes		0.72	0.32	1.18	0.09	
AGO9	AT5G21150	yes	no		0.10	0.14	-0.47	1.00	
CLSY1	AT3G42670	yes	no		1.03	0.92	0.16	0.81	
CMT2	AT4G19020	yes	yes		4.93	2.52	0.97	0.09	
CMT3	AT1G69770	no	no		5.20	1.99	1.39	0.04	
DCL4	AT5G20320	yes	no		2.55	2.40	0.09	0.88	
DDM1	AT5G66750	no	no		2.72	1.29	1.07	0.06	
DMS3	AT3G49250	yes	no		4.18	1.91	1.13	0.05	
DMS4	AT2G30280	yes	no		5.32	6.37	-0.26	0.63	
DRD1	AT2G16390	yes	no		1.62	1.56	0.06	0.93	
HEN1	AT4G20910	yes	yes		2.62	1.26	1.06	0.08	
IDP1	AT1G15910	yes	no		5.76	9.28	-0.69	0.22	
KTF1	AT5G04290	yes	no		6.48	9.31	-0.52	0.38	
LDL2	AT3G13682	yes	no		0.86	0.94	-0.14	0.84	
MET1	AT5G49160	yes	no		5.33	2.60	1.03	0.04	
MORC1	AT4G36290	yes	no		3.92	3.65	0.10	0.86	
MORC6	AT1G19100	yes	no		4.03	3.54	0.18	0.73	
NRPD1(Pol IV)	AT1G63020	yes	yes		0.94	0.73	0.37	0.56	
NRPD2/NRPE2(Pol IV/Pol V)	AT3G23780	yes	no		5.83	4.03	0.53	0.32	
NRPE9B(Pol V)	AT4G16265	yes	no		9.79	7.94	0.30	0.63	
RDM1	AT3G22680	yes	no		30.93	27.64	0.16	0.75	
RDR6	AT3G49500	yes	no		4.79	3.62	0.41	0.45	
SHH1	AT1G15215	yes	no		3.79	4.16	-0.14	0.90	
SUVH5	AT2G35160	yes	no		0.44	0.39	0.18	1.00	
SUVH6	AT2G22740	yes	no		4.99	4.43	0.17	0.77	
SUVH9	AT4G13460	yes	no		15.83	13.23	0.26	0.64	
VIM1	AT1G57820	no	no		7.45	2.77	1.43	0.01	
				Significant					log2FC>1&Pvalue<0.05
				Only in Control					log2FC>0.8&Pvalue<0.1

Supplementary Table 11 | Statistical summary of small RNA sequencing libraries and sequenced read annotation.

	Dark	Control
total reads	18829885	23336567
genome	3820103	4087642
exon_sense	73966	143556
miRNA	3165638	3182298
rRNA	9163365	12079985
repeat	3453	5222
snRNA	34643	29660
snoRNA	23294	11250
tRNA	1274481	1014403
unann	5091045	6870193

Supplementary Table 12 | Differential TE families in terms of siRNAs under extended darkness treatment and control condition.

	Genome	siRNA-increased TEs	siRNA-decreased TEs
DNA/En-Spm	356	3	5
DNA/HAT	84	0	4
DNA/MuDR	725	14	23
LINE/L1	398	6	12
LTR/Copia	514	9	26*
LTR/Gypsy	1277	134*	55*
null/SADHU	17	2	0
RC/Helitron	166	0	2
Unassigned	326	15	4

* Indicates a significant difference. Significance was calculated by hypergeometric distribution with a cut-off of 0.05.

Supplementary Data 1 | DHS-changed genes and closest peaks of differential DHSs after dark treatment (non-TE).
(in additional excel file)

Supplementary Data 2 | DHS-changed genes and closest peaks of differential DHSs after dark treatment (TE).
(in additional excel file)

Supplementary Data 3 | Comparison of DHS-changed genes between DH_WT_dark treatment_vs_control and DH_WT_dark treatment_vs_GSM847326-28.
(in additional excel file)

Supplementary Data 4 | Differentially expressed genes under extended darkness treatment and control condition.
(in additional excel file)

Supplementary Data 5 | Q-RT-PCR validation of the RNA-seq data.
(in additional excel file)

Supplementary Data 6 | The closest peaks and expression levels of 519 DHS-diminished and down-regulated genes under extended darkness.
(in additional excel file)

Supplementary Data 7 | The closest peaks and expression levels of 180 DHS-diminished and up-regulated genes under extended darkness.
(in additional excel file)

Supplementary Data 8 | Comparison of selected enriched GO terms among DHS-diminished and up-regulated genes under extended darkness, all DHS-diminished genes, and all down-regulated genes.
(in additional excel file)

Supplementary Data 9 | DHS-diminished and down-regulated genes involved in photosynthesis.
(in additional excel file)

Supplementary Data 10 | Gene list of siRNA-changed TEs under extended darkness and control condition.

(in additional excel file)
